# Genomic and clinical characteristics of *Staphylococcus aureus* isolates associated with orthopedic infections from a rural hospital, Qingdao, China

**DOI:** 10.1128/spectrum.03692-25

**Published:** 2026-03-23

**Authors:** Ying Wang, Yingdi Wang, Chao Liu, Dan Zhao, Yanxiang Cui, Xiaoxuan Guan, Guilai Jiang, Shunqian Yuan, Xinting Meng, Jianqiang Hu, Wenbo Xia, Jie Liu

**Affiliations:** 1 School of Public Health, Qingdao University 12593, Qingdao, Shandong, China; 2 Medical Department, LaiXi Municipal Hospital, Qingdao, Shandong, China; 3 Department of Clinical Laboratory, Qingdao Huangdao District Traditional Chinese Medicine Hospital 609297 , Qingdao, Shandong, China; 4 NHC Key Laboratory of Systems Biology of Pathogens, National Institute of Pathogen Biology, Chinese Academy of Medical Sciences and Peking Union Medical College 220736, Beijing, China; 5 Department of Orthopedics, Qingdao Huangdao District Traditional Chinese Medicine Hospital 609297 , Qingdao, Shandong, China; Houston Methodist, Houston, Texas, USA

**Keywords:** *Staphylococcus aureus*, orthopedic infections, clinical isolates, antimicrobial resistance, whole-genome sequencing

## Abstract

*Staphylococcus aureus* is a leading pathogen associated with orthopedic infections, particularly in resource-limited settings where clinical management is complicated by antimicrobial resistance. To characterize the phenotypic and genomic features of *S. aureus* associated with orthopedic infections in rural China, 80 isolates were recovered from inpatients at a regional hospital in Qingdao. Clinical and demographic data were integrated with colony morphology, hemolysis, antimicrobial susceptibility testing, and whole-genome sequencing. Significant clinical differences were observed between community- and hospital-acquired infections in hospitalization duration, admission diagnosis, injury cause/site, and surgical intervention. The isolates represented 23 sequence types, with ST22, ST59, and ST25 being the most prevalent. Four novel STs (ST8153, ST8154, ST9407, ST9408) were identified. They were further characterized as 19 MRSA and 61 MSSA strains. 68.8% of isolates formed whitish colonies, while all were catalase-positive. 62.5% of isolates were multidrug-resistant, showing high resistance to penicillin (88.8%), erythromycin (63.8%), and clindamycin (62.5%), but all remained susceptible to vancomycin and linezolid. Comparative genomic analysis revealed diverse profiles of antimicrobial resistance genes, virulence factors, and mobile genetic elements, whose distributions were closely associated with sequence types. Phylogenetic comparison with 515 publicly available genomes highlighted clustering of local isolates with international lineages, underscoring both global dissemination and local evolution. This study expands current knowledge on the epidemiology, resistance, and genomic diversity of *S. aureus* in orthopedic infections, identifies novel STs, and provides evidence to improve surveillance and treatment strategies in rural healthcare settings.

**IMPORTANCE:**

Orthopedic infections caused by Staphylococcus aureus remain a major clinical and public health concern, especially in rural regions where diagnostic and therapeutic capacities are limited. This study represents one of the first integrated clinical-genomic analyses of *S. aureus* from orthopedic infections in rural China. By combining patient-level data with phenotypic and genomic characterization, we reveal high rates of multidrug resistance, heterogeneous virulence and resistance gene profiles, and the emergence of novel sequence types. Comparative phylogenetic analyses demonstrate strong genetic links between rural Chinese isolates and globally disseminated lineages, emphasizing the international movement of epidemic clones. Together with antibiotic treatment data, these findings underscore the need for rational antimicrobial use, infection control, and continuous genomic surveillance. The discovery of novel lineages highlights ongoing local evolution of *S. aureus* and supports the importance of integrating genomics into orthopedic infection management and antimicrobial resistance mitigation in resource-limited settings.

## INTRODUCTION

*Staphylococcus aureus* (*S. aureus*) is a major human pathogen associated with a wide spectrum of infections, ranging from mild skin and soft tissue infections (SSTI) to life-threatening diseases, such as sepsis, endocarditis, and pneumonia (1). Compounding the clinical burden, *S. aureus* has demonstrated remarkable antimicrobial resistance (AMR) to multiple antibiotic classes, including aminoglycosides, β-lactams, fluoroquinolones, tetracyclines, and macrolides. The global spread of methicillin-resistant *S. aureus* (MRSA), along with emerging multidrug-resistant (MDR) and even extensively drug-resistant (XDR) lineages, presents a serious challenge to infection control, patient management, and public health (2, 3). In 2019, *S. aureus* ranked as the second leading pathogen responsible for AMR-associated deaths, and MRSA caused more than 100,000 deaths worldwide in one single year (4, 5). More concerning, in rural or resource-limited settings, where diagnostic infrastructure and access to advanced antimicrobial treatment are relatively constrained, the management of *S. aureus* infections is even more difficult (6).

Meanwhile, the dominant lineages of *S. aureus* have undergone dynamic shifts in both hospital and community settings (7–11). In China, sequence type (ST) 239 was the predominant hospital-acquired MRSA (HA-MRSA) clone prior to 2010, consistent with Asian and global epidemiological patterns at the time. Around 2015, ST59 emerged as the leading community-associated MRSA (CA-MRSA) lineage in China and broader Asia-Pacific region, typically associated with staphylococcal protein A (*spa*) type t437 and staphylococcal cassette chromosome *mec* (SCC*mec*) types IV or V. Over the past decade, ST59 has become increasingly prevalent in both community and hospital contexts. In contrast, the methicillin-susceptible *S. aureus* (MSSA) population has remained genetically diverse and relatively stable over time, including ST5, ST7, ST22, ST188, ST398, and others.

Among *S. aureus*-associated infections, orthopedic infection is a major clinical challenge due to the anatomical complexity of bones and joints and the impaired vascularization at infected sites, which can hinder effective antimicrobial penetration and require prolonged treatment (12, 13). Without timely and appropriate treatment, orthopedic infections often become recurrent or chronic, leading to persistent pain, joint or bone destruction, and even permanent disability or death (14, 15). In developed countries, the rate of postoperative infections following fracture surgery generally ranges between 2 and 4%, but can rise to 19% when open fractures or soft tissue contamination occur (16). Even worse, the broad and heterogeneous clinical spectra of orthopedic infections further complicate accurate diagnosis and standardized research studies (17–19).

*S. aureus* has been found to be the predominant pathogen of implant-associated and post-traumatic infections, attributable for over 60% of culture-confirmed cases worldwide. These infections are notoriously difficult to treat due to the propensity of *S. aureus* to form biofilms on implanted devices and evade host immune system (20–22). In China, the burden of trauma-related orthopedic infections has increased substantially over the past decades (23). *S. aureus* infections, particularly those caused by AMR or even MDR strains, pose urgent needs for enhanced surveillance, molecular epidemiology, and targeted intervention strategies (8). Although high-quality epidemiological and genomic studies focusing on *S. aureus*-associated orthopedic infections have been conducted in urban hospitals in China, data from rural areas remain scarce (24, 25).

To address this issue, the current study conducted a comprehensive phenotypic and genomic investigation on 80 *S. aureus* isolates from orthopedic inpatients at a regional hospital in rural Qingdao, China, with their clinical features characterized to explore potential risk factors associated with orthopedic *S. aureus* infections. Phylogenetic analyses were performed using both local and publicly available *S. aureus* genomes associated with orthopedic infections to elucidate evolutionary relationships and transmission patterns on a broad geographic scale.

## MATERIALS AND METHODS

### Study design

A total of 80 inpatients that were microbiologically confirmed with *S. aureus* infections were randomly selected from the Orthopedic Department of Qingdao Huangdao District Traditional Chinese Medicine Hospital between September 2020 and October 2024. Inclusion criteria were as follows: (i) hospitalization duration of at least 24 h; (ii) complete medical records, including clinical microbiology results obtained during hospitalization; (iii) isolation and identification of *S. aureus* as the primary causative pathogen for the orthopedic infection by the clinical laboratory; and (iv) availability of *S. aureus* isolates for genomic sequencing. Clinical samples, including wound exudates, drainage fluid, and joint effusions, were collected in accordance with the Guidelines for Molecular Testing Techniques for Personalized Medicine (Infectious Disease session, National Center for Clinical Laboratories, China, 2017). Medical charts were systematically reviewed, and relevant information was extracted using EpiData software (epidata.dk, Denmark), including demographic data, presenting symptoms, infection types, laboratory results, and antibiotic treatment regimens.

### Definition of hospital-acquired (HA) and community-associated (CA) *S. aureus*

According to the guidelines published by the International Nosocomial Infection Control Consortium and previous studies (26–29), *S. aureus* infections were classified as hospital-acquired (HA) if any of the following criteria were met: (i) infection occurred ≥48 h after admission; (ii) the patient had a history of hospitalization, surgery, or residence in healthcare-related facilities (e.g., nursing homes or other healthcare facilities) within the past 12 months; (iii) a positive *S. aureus* culture had been documented within 12 months prior to the current episode; or (iv) infection was associated with long-term orthopedic implants. Cases that did not meet any of the above criteria were defined as community-associated (CA) *S. aureus* infections.

### Culture of *S. aureus* isolates and phenotypic characterization

For each patient, only the first *S. aureus* isolate identified by the clinical laboratory was included in this study. Clinical specimens were initially cultured on blood agar plates (Babio, Jinan, China) and incubated at 37°C for 24 h. Growing colonies were subjected to Gram staining and identified using the VITEK 2 automated microbiology system (bioMérieux, Marcy-l'Étoile, France). Collected colonies were then suspended in phosphate-buffered saline (PBS; BasalMedia, Shanghai, China) and subjected to 10-fold serial dilutions. A 10 μL aliquot of each dilution was spread onto fresh agar plates for further purification and preparation of whole-genome sequencing.

Phenotypic characteristics were assessed, including colony morphology, pigmentation, hemolytic activity, and catalase production. Catalase activity was determined by applying hydrogen peroxide (Lixing, Shandong, China) directly to bacterial colonies and observing for bubble formation (30, 31). All confirmed *S. aureus* isolates were preserved in tryptic soy broth (TSB; Biosharp, Beijing, China) supplemented with 15% glycerol and stored at −80°C for subsequent analyses.

### Antimicrobial susceptibility testing

AMR profiles of the *S. aureus* isolates were determined using the VITEK 2 system following the manufacturer’s instructions. The antimicrobial panel included cefoxitin, oxacillin, penicillin, vancomycin, clindamycin, erythromycin, gentamicin, linezolid, quinupristin/dalfopristin, tetracycline, tigecycline, ciprofloxacin, levofloxacin, moxifloxacin, rifampicin, and sulfamethoxazole. The corresponding drug classes, molecular targets, and mechanism of action (MOA) are summarized in Table S1. Isolates exhibiting resistance to at least one agent in three or more antimicrobial categories were classified as MDR phenotype, whereas XDR isolates were defined as those remaining susceptible to only one or two drug categories in accordance with previously published criteria (3).

Susceptibilities to cefoxitin, penicillin, vancomycin, and linezolid were independently confirmed using the Kirby-Bauer disk diffusion method to validate the VITEK 2 results. Briefly, clinical isolates were inoculated onto Mueller-Hinton agar plates and incubated at 37°C for 18–24 h with antibiotic disks (Oxoid, Basingstoke, UK). Results were interpreted according to the Clinical and Laboratory Standards Institute (CLSI) guidelines (M100, editions 31–35, Malvern, PA, United States). *S. aureus* subsp. *aureus* Rosenbach ATCC 25,923 was used as the quality control strain.

### Whole-genome sequencing

All clinical isolates were cultured in 15 mL of TSB medium at 37°C for 12 h. Prior to whole-genome sequencing (WGS), the identity of each isolate was confirmed by 16S rRNA sequencing as previously described (32). Genomic DNA was extracted using the MagPure Bacterial DNA Kit (Magen, Guangzhou, China) following the manufacturer’s instructions. DNA concentration and integrity were assessed using a Qubit 4.0 fluorometer (Thermo Scientific, Shanghai, China) and agarose gel electrophoresis. DNA was then fragmented to an average size of 200–400 bp, followed by 3′-end adenylation, adapter ligation, and PCR amplification. Sequencing libraries were validated using Qubit and agarose gel electrophoresis and subsequently sequenced on the Illumina NovaSeq 6000 platform (Sangon Biotech, Shanghai, China). Raw reads were quality-checked with QUality Assessment Toolkit (QUAST) and CheckM v2, filtered using Trimmomatic v0.36, and assembled with SPAdes v3.15. Gaps were filled using GapFiller v1.11. Over 1.9 Gb of high-quality clean data were generated per genome, with an average sequencing depth of 100×. The assembled genome sizes ranged from 2.62 to 5.59 Mb, with a mean size of 2.95 Mb. The average guanine-cytosine content was approximately 32.0%. Genome assemblies resulted in 20 to 78 contigs per isolate, with N50 values ranging from 71,046 to 431,970 bp. Gene prediction and annotation were performed using Prokka v1.10 and the National Center for Biotechnology Information (NCBI) database.

### Genetic characterization of *S. aureus* isolates

Genetic analyses and typing were performed based on the genome assemblies from whole-genome sequencing of the 80 *S. aureus* isolates. The resistome, virulome, and mobile genetic elements (MGEs) of all isolates were identified using the Comprehensive Antibiotic Resistance Database (CARD, https://card.mcmaster.ca/), the Virulence Factor Database (VFDB, https://www.mgc.ac.cn/VFs/main.htm), and Mobile Element Finder (https://cge.food.dtu.dk/services/MobileElementFinder/), respectively. For CARD and VFDB analyses, thresholds for sequence identity, query coverage, and E-value were set at 70%, 70%, and < 1e-5, respectively. For MGE detection, thresholds were set at 90% identity, 95% coverage, and < 1e-5. Only the top-scoring hit was retained for each query (33–35). MRSA was defined by the presence of the *mecA* gene as identified by CARD and was also confirmed by phenotypic antimicrobial susceptibility testing performed in the clinical microbiology laboratory.

Multilocus sequence typing (MLST) was conducted for all 80 isolates by uploading whole-genome sequences to the *S. aureus* database of PubMLST (https://pubmlst.org/) using default parameters. STs and clonal complexes (CCs) were assigned based on the allelic profiles of seven housekeeping genes (*arcC*, *aroE*, *glpF*, *gmk*, *pta*, *tpi*, and *yqiL*). Newly identified alleles and STs were submitted to and registered in the PubMLST database (36, 37). In addition, *spa* typing and SCC*mec* typing were conducted for all 80 isolates using the Center for Genomic Epidemiology tools spaTyper and SCCmecFinder (https://cge.food.dtu.dk/services/spaTyper/, https://cge.food.dtu.dk/services/ SCCmecFinder/) with default settings applied. For SCCmecFinder, nucleotide identity and coverage thresholds were set at ≥ 90% and ≥ 60%, respectively (38, 39).

### Phylogenetic analyses of *S. aureus* isolates

A maximum likelihood (ML) phylogenetic tree was constructed based on 81 core genes identified using UBCG2 (http://leb.snu.ac.kr/ubcg2) and visualized with iTOL (https://itol.embl.de/) (40, 41). The ML tree of the 80 *S. aureus* isolates from this study was rooted using the midpoint method (42). To expand the phylogenetic context, 515 non-redundant, high-quality genomes of *S. aureus* associated with orthopedic infections were retrieved from the NCBI database and analyzed alongside the isolates of this study. *S. epidermidis* ATCC 14,990 (NZ_CP035288.1) was used as the outgroup. In addition, a minimum spanning tree (MST) was constructed based on the seven housekeeping genes. The MST was visualized using GrapeTree (https://enterobase.readthedocs.io/en/latest/grapetree/grapetree-about.html) (43).

### Statistical analyses

Statistical analyses were performed using IBM SPSS Statistics version 26. Independent-sample Student’s *t*-test, Mann-Whitney *U* test, and Kruskal-Wallis *H* test were used for quantitative variables, and χ² or Fisher’s exact tests were applied for categorical variables. A *P* value < 0.05 was considered statistically significant.

## RESULTS

### Demographic characteristics of enrolled patients

Consistent with previous studies (44), 80.0% of the enrolled patients were male (64/80), and the average age was 46.7 ± 17.6. The median hospital stay was 16.0 days (IQR 10.0, 29.7), with two exceeding 90 days. Upon admission, the most common clinical diagnoses were SSTI (31.3%, 25/80) and postoperative infection (22.5%, 18/80). The lower extremities (57.5%, 46/80) and closed injuries (56.3%, 45/80) were the most frequently affected anatomical sites and injury types, respectively. 66.3% of patients (53/80) underwent surgical intervention (Table 1).

**TABLE 1:** Demographic characteristics of enrolled patients in this study stratified by epidemiological subgroups

	Total (*n* = 80)	CA^*a*^ (*n* = 48)	HA (*n* = 32)	*P*-value	MRSA (*n* = 19)	MSSA (*n* = 61)	*P*-value	CA-MRSA (*n* = 10)	HA-MRSA (*n* = 9)	CA-MSSA (*n* = 38)	HA-MSSA (*n* = 23)	*P*-value
Gender				0.732			0.514					0.323
Male	64 (80.0%)^*b*^	39 (81.3%)	25 (78.1%)		14 (73.7%)	50 (80.2%)		9 (90.0%)	5 (55.6%)	30 (78.9%)	20 (87.0%)	
Female	16 (20.0%)	9 (18.7%)	7 (21.9%)		5 (26.3%)	11 (18.0%)		1 (10.0%)	4 (44.4%)	8 (21.1%)	3 (13.0%)	
Ages^*c*^	46.7, 17.6	46.2, 17.7	47.3, 17.8	0.776	46.5, 17.2	46.7, 17.9	0.961	42.9, 15.0	50.4, 19.5	47.1, 18.4	46.1, 17.4	0.829
Duration of hospitalization (days)^*d*^	16.0 (10.0, 29.7)	11.0 (8.0, 18.7)	28.0 (18.0, 52.5)	**<0.001**	25.0 (15.0, 43.0)	13.0 (9.0, 23.0)	**0.009**	16.0 (11.0, 26.2)	29.0 (26.5, 50.0)	10.0 (7.0, 15.7)	21.0 (15.0, 56.0)	**<0.001**
Clinical diagnosis				**<0.001**			0.348					**<0.001**
Skin and Soft Tissue Infections	25 (31.3%)	21 (43.8%)	4 (12.5%)		6 (31.6%)	19 (31.1%)		6 (60.0%)	0 (0.0%)	15 (39.5%)	4 (17.4%)	
Fracture	11 (13.8%)	0 (0.0%)	11 (34.4%)		5 (26.3%)	6 (9.8%)		0 (0.0%)	5 (55.6%)	0 (0.0%)	6 (26.1%)	
Postoperative	18 (22.5%)	15 (31.3%)	3 (9.4%)		3 (15.8%)	15 (24.6%)		3 (30.0%)	0 (0.0%)	12 (31.6%)	3 (13.0%)	
Others	26 (32.5%)	12 (25.0%)	14 (43.8%)		5 (26.3%)	21 (34.4%)		1 (10.0%)	4 (44.4%)	11 (28.9%)	10 (43.5%)	
Causes				**0.006**			0.627					**0.019**
Without obvious cause	47 (58.8%)	35 (72.9%)	12 (37.5%)		13 (68.4%)	34 (55.7%)		10 (100.0%)	3 (33.3%)	25 (65.8%)	9 (39.1%)	
Injury	19 (23.8%)	8 (16.7%)	11 (34.4%)		3 (15.8%)	16 (26.2%)		0 (0.0%)	3 (33.3%)	8 (21.1%)	8 (34.8%)	
Others	14 (17.5%)	5 (10.4%)	9 (28.1%)		3 (15.8%)	11 (18.0%)		0 (0.0%)	3 (33.3%)	5 (13.2%)	6 (26.1%)	
Injury sites				**0.020**			0.313					0.053
Upper limbs	19 (23.8%)	15 (31.3%)	4 (12.5%)		6 (31.6%)	13 (21.3%)		5 (50.0%)	1 (11.1%)	10 (26.3%)	3 (13.0%)	
Lower limbs	46 (57.5%)	28 (58.3%)	18 (56.3%)		8 (42.1%)	38 (62.3%)		5 (50.0%)	3 (33.3%)	23 (60.5%)	15 (65.2%)	
Trunk	5 (6.3%)	3 (6.3%)	2 (6.3%)		1 (5.3%)	4 (6.6%)		0 (0.0%)	1 (11.1%)	3 (7.9%)	1 (4.3%)	
Multiple body parts	10 (12.5%)	2 (4.2%)	8 (25%)		4 (21.1%)	6 (9.8%)		0 (0.0%)	4 (44.4%)	2 (5.3%)	4 (17.4%)	
Types of injuries				1.000			0.600					0.920
Closed Injury	45 (56.3%)	27 (56.3%)	18 (56.3%)		12 (63.2%)	33 (54.1%)		6 (60.0%)	6 (66.7%)	21 (55.3%)	12 (52.2%)	
Open Injury	35 (43.8%)	21 (43.8%)	14 (43.8%)		7 (36.8%)	38 (45.9%)		4 (40.0%)	3 (33.3%)	17 (44.7%)	11 (47.8%)	
Surgical intervention				**0.001**			1.000					**0.010**
Yes	53 (66.3%)	25 (52.1%)	28 (87.5%)		13 (68.4%)	40 (65.6%)		5 (50.0%)	8 (88.9%)	20 (52.6%)	20 (87.0%)	
No	27 (33.8%)	23 (47.9%)	4 (12.5%)		6 (31.6%)	21 (34.4%)		5 (50.0%)	1 (11.1%)	18 (47.4%)	3 (13.0%)	
Clinical presentations												
Purulent exudate	16 (20.0%)	12 (25.0%)	4 (12.5%)	0.255	2 (10.5%)	14 (23.0%)	0.333	1 (10.0%)	1 (11.1%)	11 (28.9%)	3 (13.0%)	0.419
Redness	46 (57.5%)	34 (70.8%)	12 (37.5%)	**0.005**	10 (52.6%)	36 (59.0%)	0.791	7 (70.0%)	3 (33.3%)	27 (71.1%)	9 (39.1%)	0.033
Swelling	50 (62.5%)	29 (60.4%)	21 (65.6%)	0.814	12 (63.2%)	38 (62.3%)	1.000	5 (50.0%)	7 (77.8%)	24 (63.2%)	14 (60.9%)	0.708
Bleeding	8 (10.0%)	4 (8.3%)	4 (12.5%)	0.707	0 (0.0%)	8 (13.1%)	0.118	0 (0.0%)	0 (0.0%)	4 (10.5%)	4 (17.4%)	0.432
Pain	72 (90.0%)	44 (91.7%)	28 (87.5%)	0.707	16 (84.2%)	56 (91.8%)	0.386	8 (80.0%)	8 (88.9%)	36 (94.7%)	20 (87.0%)	0.291
Malformation	9 (11.3%)	2 (4.2%)	7 (21.9%)	**0.026**	1 (5.3%)	8 (13.1%)	0.446	0 (0.0%)	1 (11.1%)	2 (5.3%)	6 (26.1%)	0.063
Restricted activity	45 (56.3%)	26 (54.2%)	19 (59.4%)	0.818	9 (47.4%)	36 (59.0%)	0.433	3 (30.0%)	6 (66.7%)	23 (60.5%)	13 (56.5%)	0.340
Tissues exposed	1 (1.3%)	0 (0.0%)	1 (3.1%)	0.400	0 (0.0%)	1 (1.6%)	1.000	0 (0.0%)	0 (0.0%)	0 (0.0%)	1 (4.3%)	0.525
Contaminated wound	3 (3.8%)	0 (0.0%)	3 (9.4%)	0.060	0 (0.0%)	3 (4.9%)	1.000	0 (0.0%)	0 (0.0%)	0 (0.0%)	3 (13.0%)	0.093
Rupture	27 (33.8%)	18 (37.5%)	9 (28.1%)	0.472	6 (31.6%)	21 (34.4%)	1.000	5 (50.0%)	1 (11.1%)	13 (34.2%)	8 (34.8%)	0.380
Exudate	33 (41.3%)	21 (43.8%)	12 (37.5%)	0.647	9 (47.4%)	24 (39.3%)	0.599	5 (50.0%)	4 (44.4%)	16 (42.1%)	8 (34.8%)	0.849
Elevated skin temperature	25 (31.3%)	19 (39.6%)	6 (18.8%)	0.084	5 (26.3%)	20 (12.8%)	0.778	3 (30.0%)	2 (22.2%)	16 (42.1%)	4 (17.4%)	0.231
Subcutaneous hemorrhage	8 (10.0%)	4 (8.3%)	4 (12.5%)	0.707	1 (5.3%)	7 (11.5%)	0.672	0 (0.0%)	7 (11.5%)	4 (10.5%)	3 (13.0%)	0.807
Effusion	7 (8.8%)	5 (10.4%)	2 (6.3%)	0.696	0 (0.0%)	7 (11.5%)	0.145	0 (0.0%)	0 (0.0%)	5 (13.2%)	2 (8.7%)	0.649
Poor circulation	5 (6.3%)	3 (6.3%)	2 (6.3%)	1.000	0 (0.0%)	5 (8.2%)	0.332	0 (0.0%)	0 (0.0%)	3 (7.9%)	2 (8.7%)	1.000

^*a*^ CA, community-acquired; HA, hospital-acquired; MRSA, methicillin-resistant *Staphylococcus aureus*; MSSA, methicillin-susceptible *Staphylococcus aureus*.

^*b*^ Percentage calculated based on the total number of cases within each epidemiological subgroup.

^*c*^ Age is presented as mean ± standard deviation.

^*d*^ Duration of hospitalization is presented as the interquartile range, P50 (P25, P75).

Patients were categorized into CA and HA *S. aureus* infection groups (48 vs 32), as well as MRSA and MSSA groups (19 vs 61). Significant differences between CA and HA groups were observed in hospitalization duration (*P* < 0.001), clinical diagnosis (*P* < 0.001), infection causes (*P* = 0.006), injury site (*P* = 0.020), surgical intervention (*P* = 0.001), and clinical manifestations, such as redness (*P* = 0.005) and malformation (*P* = 0.026). In the CA group, SSTI (43.8%, 21/48) and postoperative infection (31.3%, 15/48) were the predominant diagnoses, whereas fractures were mostly common in the HA group (34.4%, 11/32). Surgeries were performed in 87.5% (28/32) of HA cases, compared to 52.1% (25/48) in the CA group. Only hospitalization duration demonstrated a significant difference between the MRSA and MSSA groups (*P* = 0.009). Patients were further stratified into four epidemiological subgroups: CA-MRSA (12.5%, 10/80), HA-MRSA (11.25%, 9/80), CA-MSSA (47.5%, 38/80), and HA-MSSA (28.75%, 23/80), among which hospitalization (*P* < 0.001), diagnosis (*P* < 0.001), causes (*P* = 0.019), and surgical treatment (*P* = 0.010) varied significantly (Table 1).

### Phenotypic characterization of colony morphology, hemolytic characteristics, and catalase activity

Colony morphology, hemolytic patterns, and catalase activity were evaluated. Fifty-five isolates (68.8%) formed whitish colonies on blood agar, indicating reduced or absent golden pigmentation compared with the typical *S. aureus* phenotype. This observation was recorded as a phenotypic characteristic only, without implying altered staphyloxanthin (STX) biosynthesis or regulation (45). The remaining 25 yellowish colonies included 20 CA and 5 HA isolates, or 8 MRSA and 17 MSSA isolates. Overall, 44 whitish (80.0%, 44/55) and 18 yellowish isolates (72.0%, 18/25) displayed β-hemolysis (Fig. S1), distributed as 36 CA and 26 HA isolates or 14 MRSA and 48 MSSA isolates. Most MSSA isolates were whitish (72.1%, 44/61) and β-hemolytic (78.7%, 48/61). Fifteen isolates (5 CA and 10 HA, or 5 MRSA and 10 MSSA) exhibited α-hemolytic activity. Only three whitish MSSA isolates were γ-hemolytic. Additionally, all isolates tested positive for catalase activity (Table S2).

### Whole-genome sequencing and phylogenetic analysis

#### Phylogenetic analysis of the 80 *S. aureus* isolates along with publicly available genomes from the NCBI database

**Fig 1:**
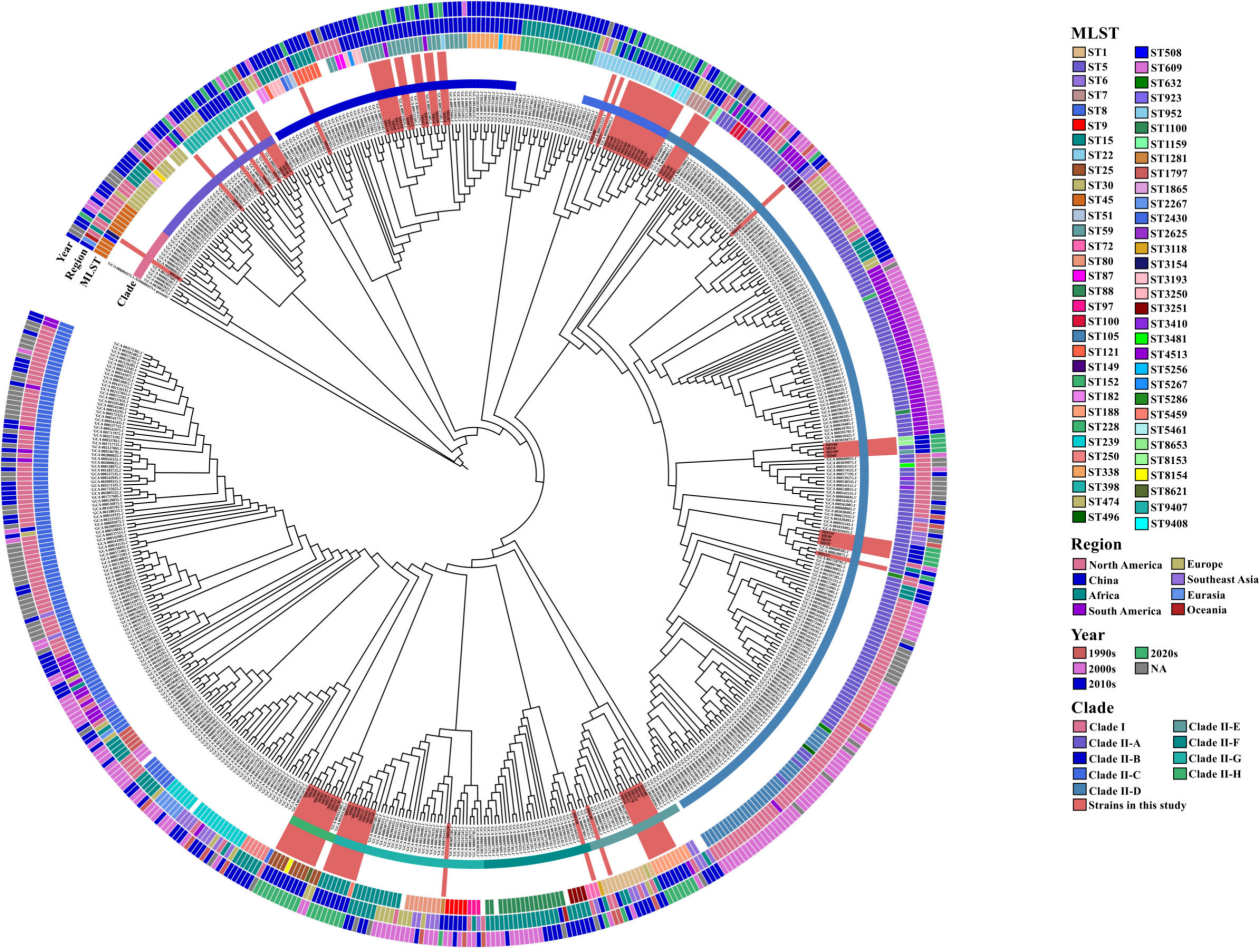
Maximum likelihood phylogenetic tree of the 80 *S. aureus* isolates and 515 publicly available genomes from the NCBI database. Sequence type (ST), geographic origin, collection year, clade, and subclade classifications (Clade II-A to Clade II-H) are annotated using colored rings. The 80 isolates from this study are highlighted with a red background fill.

Five hundred fifteen *S. aureus* genomes associated with orthopedic infections, collected between 1933 and 2018 across 41 countries, were included to construct a ML phylogenetic tree with the reported 80 isolates. Two hundred twenty genomes (42.7%) originated from the United States, 118 (22.9%) from China, and 103 (20.0%) from Africa. The most prevalent STs were ST5, ST8, and ST105 (144, 102, and 31 genomes each) (Fig. 1).

The tree comprised two major clades with distinct temporal and geographic distributions. Clade I included 13 isolates, most of which were ST45 from Africa, Australia, and the U.S.A., alongside our isolate HD169, as the only one from China. In contrast, Clade II contained the majority of genomes and was further divided into 15 subclades, within which the remaining 79 isolates from this study were unevenly distributed across Clades II-A to II-H (Fig. 1; see supplementary text). The previously reported genomes from China distributed in Clades II-A (*n* = 4), II-B (*n* = 27), II-F (*n* = 1), and II-G (*n* = 5), while the Chinese isolates in Clades II-C, II-D, II-E, and II-H were exclusively from the current study. Our isolates clustered with the other Chinese genomes in Clades II-A and II-B while forming separate clusters in Clades II-F and II-G. Interestingly, despite the inclusion of genomes from diverse geographic regions, isolates from China, both previously reported ones and those from this study, tended to cluster together at various phylogenetic levels (Fig. 1).

#### MLST typing and distribution of *S. aureus* isolates

**Fig 2:**
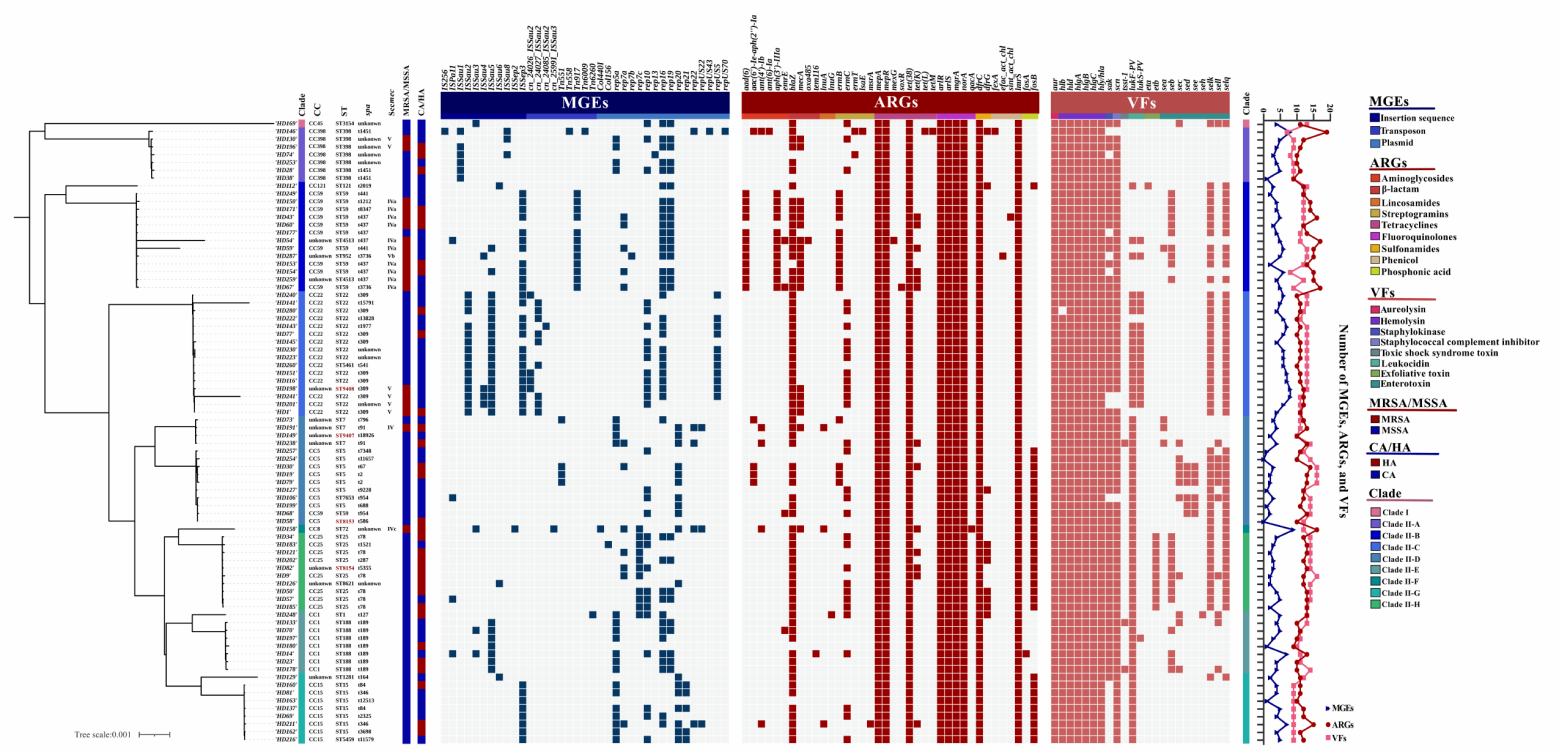
Phylogenetic structure, genotypic, and epidemiological characteristics of the 80 *S. aureus* isolates. Annotations include clonal complex (CC), ST, *spa* type, SCC*mec* type, and epidemiological category (MRSA vs MSSA; CA vs HA). Novel STs are marked in red. The presence and counts of mobile genetic elements (MGEs), antimicrobial resistance genes (ARGs), and virulence factors (VFs) for each isolate are shown. Subclade assignments correspond to the color scheme used in Fig. 1. The tree scale bar represents 0.1% nucleotide sequence divergence.

MLST typing of the 80 *S. aureus* isolates identified 23 distinct STs. The most prevalent ST was ST22 (17.5%, 14/80), followed by ST59 (13.8%, 11/80) and ST25 (10.0%, 8/80). ST5, ST15, ST188, and ST398 each accounted for seven isolates (8.8% of 80) (Fig. S2A). Of the most prevalent STs, all 14 ST22 isolates exhibited β-hemolysis, and 85.7% (12/14) were whitish; all ST25 isolates (*n* = 8) were whitish MSSA, while all ST5 isolates (*n* = 7) were β-hemolytic MSSA. All 15 CC22 isolates were β-hemolytic, with 80.0% (12/15) exhibiting whitish pigmentation (Fig. 2; Table S2).

Statistical analysis revealed that ST25 (*n* = 8) and ST59 (*n* = 11) isolates were significantly associated with whitish and yellowish pigmentation, respectively (*P* = 0.005), while HA isolates appeared more frequently whitish and CA strains were more yellowish (*P* = 0.014). Specifically, CA-MRSA isolates were significantly associated with yellowish pigmentation compared to other groups (*P* = 0.022). No statistically significant differences were observed between isolates with α- and β-hemolytic phenotypes (data not shown).

**Fig 3:**
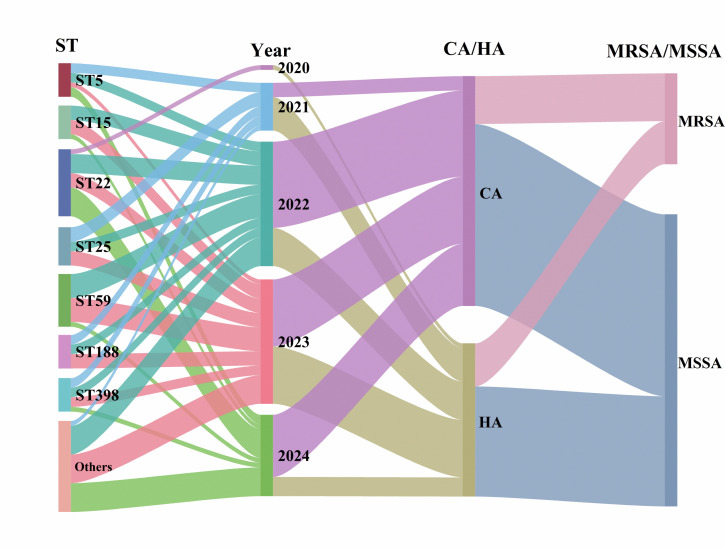
Distribution of the 80 *S. aureus* isolates showing correlations among STs, year of collection, and epidemiological subgroups (CA vs HA; MRSA vs MSSA).

In terms of epidemiological classification, CA strains predominated in 2024 (76.5%, 13/17), while HA strains were more frequently identified in 2023 (46.2%, 12/26). Similarly, MRSA and MSSA isolates were more prevalent in 2023 (34.6%, 9/26) and 2024 (82.4%, 14/17), respectively. Overall, CA-MSSA was the most common subtype throughout the study period (2020–2024), peaking in 2022 with 15 isolates (38 in total) (Fig. 3; Fig. S2B). Among CA-MRSA, HA-MRSA, CA-MSSA, and HA-MSSA subgroups, only ST59 distribution was statistically significant (*P* = 0.040).

Additionally, four *S*. *aureus* isolates were assigned to novel ST types, i.e., ST8153 (HD58, novel allele: *glpF* 987), ST8154 (HD82, *glpF* 988), ST9407 (HD149, new ST), and ST9408 (HD198, *yqiL* 1202) (Fig. 2).

#### Phylogenetic analysis and genomic characterization of the 80 *S. aureus* isolates

Similar to the clade and subclade structure in Fig. 1, all isolates were classified into nine phylogenetic clades, i.e., Clade I and Clade II-A through II-H, with no clear correlation between phylogenetic grouping and isolation year, or CA/ HA origin, although isolates belonging to the same ST tended to cluster closely (Fig. 2).

Whole-genome analysis of the 80 *S. aureus* isolates revealed genomic clustering patterns and a relatively sporadic distribution of antimicrobial resistance genes (ARGs), virulence factors (VFs), or mobile genetic elements (MGEs) across lineages. Seventy isolates were assigned to 36 different *spa* types, with the most prevalent being t309 (11.3%, 9/80), t189 (8.8%, 7/80), and t437 (8.8%, 7/80). All t309 isolates were β-hemolytic. All t189 isolates were MSSA-ST188 harboring Panton-Valentine leukocidin (PVL) gene *lukF-PV*. t437 was the most common in MRSA (*n* = 6), all of which were associated with SCC*mec* type IVa. The rest of the 10 isolates could not be assigned a *spa* type due to untypeable sequence data. SCC*mec* typing identified only type IV (*n* = 12) and V (*n* = 7) among the 19 MRSA strains. Both types are typically associated with CA-MRSA (46, 47) but were inconsistently distributed among 10 CA-MRSA and 9 HA-MRSA isolates in this study (Fig. 2; Table S2).

#### Virulome predicted by whole-genome analysis

Twenty-three distinct VFs were identified (Fig. 2). All isolates carried the aureolysin-encoding gene (*aur*) and hemolysin-associated genes, including *hld*, *hlgABC*, and *hly/hla*. The *hlb* gene was present in all isolates except HA-MSSA-ST22 HD280, which was β-hemolytic despite lacking *hlb*, while hemolytic phenotypes varied among α-, β-, and γ-types (Table S2). The immune evasion cluster (IEC) genes, particularly *sak* and *scn*, which are associated with evasion of host immune responses, were identified in 64 and 76 isolates, respectively. Notably, all *sak*-positive strains also carried *scn*, but not all *scn*-positive isolates harbored *sak*, particularly ST15 isolates of Clade II-G clustered at the bottom of Fig. 2 (48). Fifty-nine isolates carried *lukF-PV*, which were assigned to ST5, ST15, ST22, ST25, and ST188, clustered in Clade II-C through II-H. Seventeen isolates carried both *lukF-PV* and *lukS-PV*, the majority of which was ST22 (70.6%, 12/17) within Clade II-C.

The toxic shock syndrome toxin-1 (TSST-1) encoding gene *tsst-1* was exclusively identified in three phylogenetically distant isolates (HD133, HD178, and HD238). The corresponding patients presented with infectious arthritis or open wound infections. Notably, the patient from whom HD238 was isolated had the most *S. aureus*-positive cultures in this cohort, supporting previous reports that TSST-1 is associated with severe *S. aureus* infections (1).

Regarding the exfoliative toxin genes *eta* and *etb*, only CA-MSSA-ST121 HD112 harbored *eta*, whereas 10 MSSA isolates, predominantly ST25 (8/10), carried *etb* and formed a distinct cluster, Clade II-H. The enterotoxin gene *seb* was mainly detected in ST59 (*n* = 8) and ST25 (*n* = 7), which were phylogenetically clustered in Clades II-B and II-H, respectively. Additionally, most isolates within Clades II-B, II-C, II-D, and II-H, predominately ST59, ST22, ST5, and ST25, concurrently carried *selk* and *selq*, encoding the precursors of enterotoxins K and G, respectively. The other *sec*, *sed*, *see*, and *sell* were primarily concentrated in **Clade II-D**, with ST5 being dominant (4/11, 4/6, 4/6, and 4/11 isolates, respectively) (Fig. 2).

#### Other pathogenic metrics predicted by whole-genome analysis

**Fig 4:**
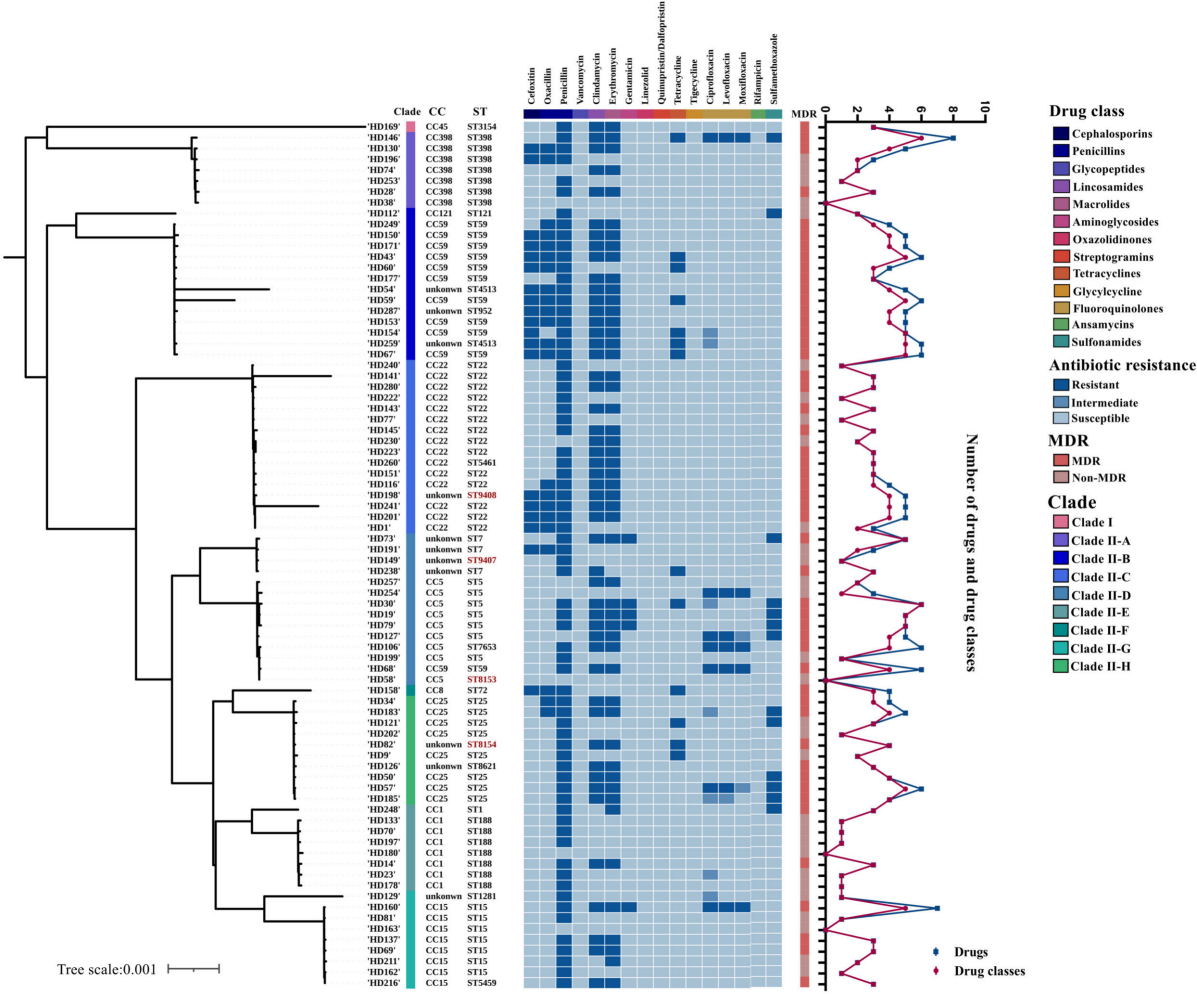
Antimicrobial susceptibility profiles of the 80 *S. aureus* isolates ordered according to phylogenetic relationships. Each isolate is annotated with its CC and ST, with the four novel STs highlighted in red. The numbers of antibiotics and antimicrobial classes to which each isolate showed resistance are plotted. MDR, multidrug-resistant.

Thirty-seven MGEs were predicted, comprising 11 insertion sequences (ISs), 9 transposons (Tn), and 17 plasmid-related elements (Fig. 2). The most prevalent ISs were IS*Sep3*, IS*Sau5*, and IS*Sau2* (33, 25, and 15 each), which were mainly enriched in Clade II-C (ST22; 12, 14, and 13). IS*Sau1* was identified in six ST398 isolates from Clade II-A, consistent with previous reports linking IS*Sau1* to CC398 lineages (49). IS*Sau3* was detected in four phylogenetically scattered isolates (CA-MSSA-ST188 HD14, CA-MSSA-ST188 HD70, HA-MRSA-ST72 HD158, and CA-MSSA-ST3154 HD169), all resistant to penicillin. Notably, HD158 was resistant to all tested β-lactams (cefoxitin, oxacillin, and penicillin) (Fig. 2 and 4; Table S3) (50). IS*Sau4* was present in four CA-MRSA isolates carrying SCC*mec* V, which was reported to facilitate horizontal gene transfer of AMR determinants (51). Statistical analysis revealed that MRSA isolates were more likely to harbor IS*Sep3* (*P* = 0.008).

**TABLE 2:** Comparative analysis of antimicrobial resistance profiles among the 80 *S. aureus* isolates^*b*^

Antibiotics^*a*^	Total (*n* = 80)	CA (*n* = 48)	HA (*n* = 32)	*P*-value	MRSA (*n* = 19)	MSSA (*n* = 61)	*P*-value	Ca-mrsa (*n* = 10)	Ha-mrsa (*n* = 9)	Ca-mssa (*n* = 38)	Ha-mssa (*n* = 23)	*P*-value
Cefoxitin				0.292			**<0.001**					**<0.001**
Susceptible	61 (75.0%)	38 (79.2%)	23 (71.9%)		0 (0.0%)	61 (100.0%)		0 (0.0%)	0 (0.0%)	38 (100.0%)	23 (100.0%)	
Intermediate	0 (0.0%)	0 (0.0%)	0 (0.0%)		0 (0.0%)	0 (0.0%)		0 (0.0%)	0 (0.0%)	0 (0.0%)	0 (0.0%)	
Resistant	19 (25.0%)	10 (20.8%)	9 (28.1%)		19 (100.0%)	0 (0.0%)		10 (100.0%)	9 (100.0%)	0 (0.0%)	0 (0.0%)	
Oxacillin				0.919			**<0.001**					**<0.001**
Susceptible	58 (72.5%)	35 (72.9%)	23 (71.9%)		1 (5.3%)	57 (93.4%)		0 (0.0%)	1 (11.1%)	35 (92.1%)	22 (95.7%)	
Intermediate	0 (0.0%)	0 (0.0%)	0 (0.0%)		0 (0.0%)	0 (0.0%)		0 (0.0%)	0 (0.0%)	0 (0.0%)	0 (0.0%)	
Resistant	22 (27.5%)	13 (27.1%)	9 (28.1%)		18 (94.7%)	4 (6.6%)		10 (100.0%)	8 (88.9%)	3 (7.9%)	1 (4.3%)	
Penicillin				0.942			0.079					0.502
Susceptible	9 (11.3%)	6 (12.5%)	3 (9.4%)		0 (0.0%)	9 (14.8%)		0 (0.0%)	0 (0.0%)	6 (15.8%)	3 (13.0%)	
Intermediate	0 (0.0%)	0 (0.0%)	0 (0.0%)		0 (0.0%)	0 (0.0%)		0 (0.0%)	0 (0.0%)	0 (0.0%)	0 (0.0%)	
Resistant	71 (88.8%)	42 (87.5%)	29 (90.6%)		19 (100.0%)	52 (85.2%)		10 (100.0%)	9 (100.0%)	32 (84.2%)	20 (87.0%)	
Clindamycin				0.098			0.249					**0.024**
Susceptible	30 (37.5%)	14 (29.2%)	16 (50.0%)		5 (26.3%)	25 (41.0%)		0 (0.0%)	5 (55.6%)	14 (36.8%)	11 (47.8%)	
Intermediate	0 (0.0%)	0 (0.0%)	0 (0.0%)		0 (0.0%)	0 (0.0%)		0 (0.0%)	0 (0.0%)	0 (0.0%)	0 (0.0%)	
Resistant	50 (62.5%)	34 (70.8%)	16 (50.0%)		14 (73.7%)	36 (59.0%)		10 (100.0%)	4 (44.4%)	24 (63.2%)	12 (52.2%)	
Erythromycin				0.154			0.302					**0.034**
Susceptible	29 (36.3%)	14 (29.2%)	15 (46.9%)		5 (26.3%)	24 (39.3%)		0 (0.0%)	5 (55.6%)	14 (36.8%)	10 (43.5%)	
Intermediate	0 (0.0%)	0 (0.0%)	0 (0.0%)		0 (0.0%)	0 (0.0%)		0 (0.0%)	0 (0.0%)	0 (0.0%)	0 (0.0%)	
Resistant	51 (63.7%)	34 (70.8%)	17 (53.1%)		14 (73.7%)	37 (60.7%)		10 (100.0%)	4 (44.4%)	24 (63.2%)	13 (56.5%)	
Gentamicin				0.384			0.332					0.486
Susceptible	75 (93.8%)	46 (95.8%)	29 (90.6%)		19 (100.0%)	56 (91.8%)		10 (100.0%)	9 (100.0%)	36 (94.7%)	20 (87.0%)	
Intermediate	0 (0.0%)	0 (0.0%)	0 (0.0%)		0 (0.0%)	0 (0.0%)		0 (0.0%)	0 (0.0%)	0 (0.0%)	0 (0.0%)	
Resistant	5 (6.3%)	2 (4.2%)	3 (9.4%)		0 (0.0%)	5 (8.2%)		0 (0.0%)	0 (0.0%)	2 (5.3%)	3 (13.0%)	
Tetracycline				**0.019**			**0.015**					**0.002**
Susceptible	67 (83.8%)	44 (91.7%)	23 (71.9%)		12 (63.2%)	55 (90.2%)		7 (70.0%)	5 (55.6%)	37 (97.4%)	18 (78.3%)	
Intermediate	0 (0.0%)	0 (0.0%)	0 (0.0%)		0 (0.0%)	0 (0.0%)		0 (0.0%)	0 (0.0%)	0 (0.0%)	0 (0.0%)	
Resistant	13 (16.3%)	4 (8.3%)	9 (28.1%)		7 (36.8%)	6 (9.8%)		3 (30.0%)	4 (44.4%)	1 (2.6%)	5 (21.7%)	
Ciprofloxacin				0.275			0.363					0.540
Susceptible	66 (82.5%)	39 (81.3%)	27 (84.4%)		17 (89.5%)	49 (80.3%)		9 (90.0%)	8 (88.9%)	30 (78.9%)	19 (86.2%)	
Intermediate	7 (8.8%)	3 (6.3%)	4 (12.5%)		2 (10.5%)	5 (8.2%)		1 (10.0%)	1 (11.1%)	2 (5.3%)	3 (13.0%)	
Resistant	7 (8.8%)	6 (12.5%)	1 (3.1%)		0 (0.0%)	7 (11.5%)		0 (0.0%)	0 (0.0%)	6 (15.8%)	1 (4.3%)	
Levofloxacin				0.162			0.380					0.390
Susceptible	72 (90.0%)	42 (87.5%)	30 (93.8%)		19 (100.0%)	53 (86.9%)		10 (100.0%)	9 (100.0%)	32 (84.2%)	21 (91.3%)	
Intermediate	1 (1.3%)	0 (0.0%)	1 (3.1%)		0 (0.0%)	1 (1.6%)		0 (0.0%)	0 (0.0%)	0 (0.0%)	1 (4.3%)	
Resistant	7 (8.8%)	6 (12.5%)	1 (3.1%)		0 (0.0%)	7 (11.5%)		0 (0.0%)	0 (0.0%)	6 (15.8%)	1 (4.3%)	
Moxifloxacin				0.460			0.460					0.775
Susceptible	73 (91.3%)	42 (87.5%)	31 (96.9%)		19 (100.0%)	54 (88.5%)		10 (100.0%)	9 (100.0%)	32 (84.2%)	22 (95.7%)	
Intermediate	2 (2.5%)	2 (4.2%)	0 (0.0%)		0 (0.0%)	2 (3.3%)		0 (0.0%)	0 (0.0%)	2 (5.3%)	0 (0.0%)	
Resistant	5 (6.3%)	4 (8.3%)	1 (3.1%)		0 (0.0%)	5 (8.2%)		0 (0.0%)	0 (0.0%)	4 (10.5%)	1 (4.3%)	
Sulfamethoxazole				0.621			0.065					0.179
Susceptible	67 (83.8%)	41 (85.4%)	26 (81.3%)		19 (100.0%)	48 (78.7%)		10 (100.0%)	9 (100.0%)	31 (81.6%)	17 (73.9%)	
Intermediate	0 (0.0%)	0 (0.0%)	0 (0.0%)		0 (0.0%)	0 (0.0%)		0 (0.0%)	0 (0.0%)	0 (0.0%)	0 (0.0%)	
Resistant	14 (17.5%)	7 (14.6%)	6 (18.8%)		0 (0.0%)	13 (21.3%)		0 (0.0%)	0 (0.0%)	7 (18.4%)	6 (26.1%)	

^*a*^ All 80 isolates were susceptible to vancomycin, linezolid, quinupristin/dalfopristin, tigecycline, and rifampicin which were not indicated.

^*b*^ Bold numbers indicate statistically significant values.

Tn917 was the most common predicted transposon (*n* = 12), predominantly associated with ST59 (*n* = 9, Clade II-B), which functions efficiently in Gram-positive genera *Bacillus*, *Staphylococcus*, and *Streptococcus* (52). The most frequently identified plasmid replication initiator genes were *rep16*, *rep10*, and *rep19* (42, 29, and 26 each), consistent with prior findings from *S. aureus* isolates in retail meat samples from Europe and China (53, 54). Since no complete transposon or plasmid sequences were detected, further structural or functional analyses of Tn and *rep* genes were not pursued (Fig. 2).

#### Genetic metrics of the isolates with newly assigned sequence types

**Fig 5:**
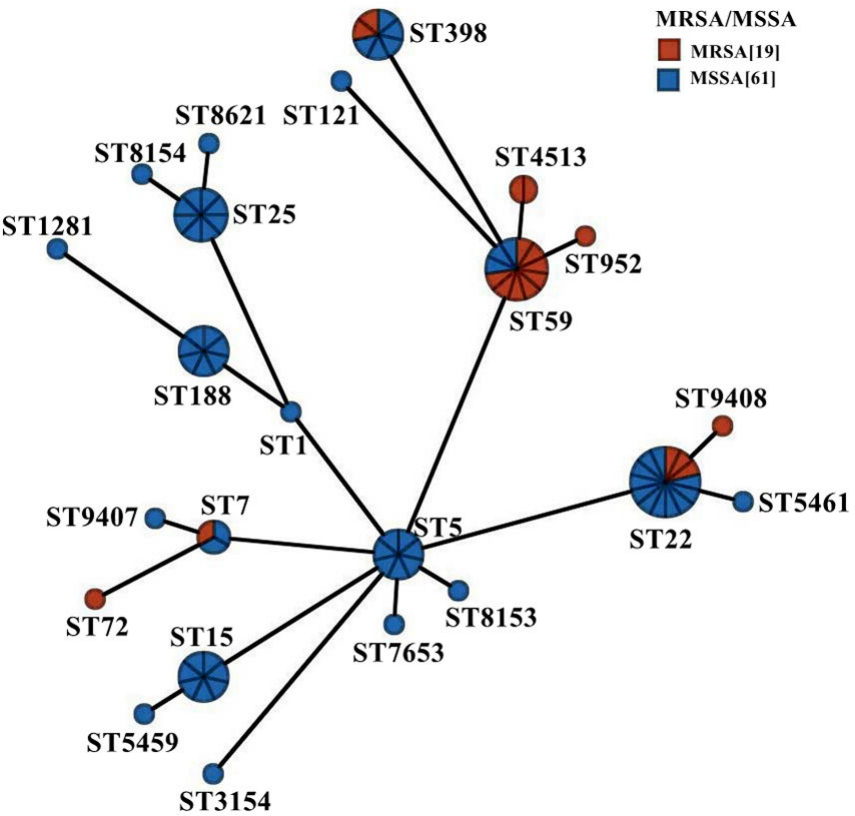
Minimum spanning tree of the 80 *S. aureus* isolates based on sequence types and MRSA/MSSA classification. Each solid circle represents a distinct ST, with circle size proportional to the number of isolates. Connecting lines denote the genetic distance between STs.

The four isolates of new STs were phylogenetically dispersed and clustered with ST22 (HD198, ST9408, CA-MRSA-t309-V-β-hemolytic), ST7 (HD149, ST9407, CA-MSSA-t18926-NA-β-hemolytic), ST5 (HD58, ST8153, HA-MSSA-t586-NA-β-hemolytic), and ST25 (HD82, ST8154, HA-MSSA-t5355-NA-γ-hemolytic), respectively, but distant from ST398 and ST59 (Fig. 2 and 5).

All four isolates harbored genes encoding aureolysin (*aur*), hemolysin (*hlb*, *hld*, *hlgA*BC, and *hly/hla*), IEC (*sak* and *scn*), leukotoxin (*lukF-PV*), and nine core ARGs (*mepA*, *mepR*, *tet (38*), *arlR*, *arlS*, *mgrA*, *norA*, *dfrC*, and *lmrS*). HD198 owned the highest number of MGEs (*n* = 8) and total predicted genetic elements (*n* = 33, including 12 ARGs and 13 VFs). HD82 carried the most ARGs (*n* = 13) and VFs (*n* = 14, including genes encoding aureolysin, hemolysins, IEC, enterotoxins, and *lukF-PV*). Both HD198 and HD82 were identified as MDR isolates. However, no complete concordance between the phenotypic and genotypic AMR profiles was observed with any of the four isolates (Fig. 2 and 4; Table S3).

### Phenotypic/genotypic antimicrobial resistances and antibiotic treatments

#### Phenotypic antimicrobial resistance

Overall, CA and HA *S. aureus* isolates exhibited comparable AMR profiles. Penicillin (88.8%, 71/80), erythromycin (63.8%, 51/80), and clindamycin (62.5%, 50/80) showed the highest resistance rates across all isolates (Fig. 4; Table 2). As expected, MRSA isolates displayed uniformly high resistance to β-lactams, with all resistant to penicillin (100%, 19/19) and 94.7% resistant to oxacillin (18/19; one isolate showed borderline minimum inhibitory concentration). In contrast, MSSA isolates showed lower oxacillin resistance (6.6%, 4/61) but similarly high resistance to penicillin (85.2%, 52/61), erythromycin (60.7%, 37/61), and clindamycin (59.0%, 36/61). All 10 CA-MRSA strains were resistant to oxacillin, penicillin, clindamycin, and erythromycin, whereas tetracycline resistance was only seen in three CA-MRSA isolates, which were not prescribed to any patient. When comparing infection origins, CA isolates showed slightly higher resistance to erythromycin and clindamy (both 70.8%, 34/48) than HA isolates (53.1%, 17/32; 50.0%, 16/32), although not statistically significant (Tables 2 and 3).

All 80 isolates remained fully susceptible to vancomycin, linezolid, quinupristin/dalfopristin, tigecycline, and rifampicin, none of which were administered except vancomycin. All 19 MRSA strains were susceptible to gentamicin, the three fluoroquinolones (ciprofloxacin, levofloxacin, and moxifloxacin), and sulfamethoxazole (Fig. 4; Tables 2 and 3). Significant differences were observed in oxacillin resistance across the four epidemiological subgroups (*P* < 0.001). Clindamycin resistance differed significantly between CA-MRSA and HA-MRSA and between CA-MRSA and HA-MSSA (*P* = 0.024) (Table 2). No overall significance was found when all antibiotic susceptibility profiles were analyzed collectively.

#### Characterization of multidrug-resistant isolates

Based on established definitions (3), 50 of the 80 isolates (62.5%) were classified as MDR, comprising 10 CA-MRSA, 22 CA-MSSA, 6 HA-MRSA, and 12 HA-MSSA. These MDR isolates were distributed across the phylogenetic tree without forming distinct lineage-specific clusters. They accounted for most resistance phenotypes, ranging from 69.0% of penicillin (49/71) to 100% of gentamicin (5/5) with the most frequent resistance observed to penicillin (*n* = 49), clindamycin (*n* = 47), and erythromycin (*n* = 47) (Fig. 4; Table S3). CA isolates demonstrated significantly higher resistance to clindamycin and erythromycin than HA (both *P* = 0.042), while MRSA isolates were more resistant to tetracycline (*P* = 0.029) but susceptible to sulfamethoxazole (*P* = 0.027).

Only four MDR isolates (CA-MSSA-ST59 HD68, CA-MSSA-ST7653 HD106, CA-MSSA-ST398 HD146, and HA-MSSA-ST15 HD160) exhibited resistance to all three fluoroquinolones tested. Among the most extensively resistant isolates, HA-MSSA-ST5 HD30 and CA-MSSA-ST398 HD146 displayed resistance to six of the eight antibiotic classes examined. In particular, HD146 was resistant to eight antibiotics representing six classes (penicillin, clindamycin, erythromycin, tetracycline, ciprofloxacin, levofloxacin, moxifloxacin, and sulfamethoxazole) and phylogenetically distinct from the aforementioned highly resistant isolates (Fig. 4; Table S3).

Comparative analysis among the seven most prevalent STs revealed the following proportions of MDR isolates: ST59 (100%, 11/11), ST22 (64.3%, 9/14), ST25 (62.5%, 5/8), ST5 (57.1%, 4/7), ST15 (42.9%, 3/7), ST398 (42.9%, 3/7), and ST188 (14.3%, 1/7). ST59 exhibited significantly higher resistance to cefoxitin compared with the other STs (*P* < 0.001), consistent with its high proportion of MRSA (8/11). ST59 strains also showed significantly higher clindamycin resistance compared to ST398 (*P* = 0.044). When considering antibiotics that inhibit protein synthesis as a group, ST59 isolates demonstrated greater resistance than ST188 (*P* = 0.005). Furthermore, ST5 and ST25 strains were significantly more resistant to sulfamethoxazole than those of ST22 (*P* < 0.001). Collectively, ST59 was strongly associated with the multidrug-resistant phenotype (*P* = 0.008).

#### Antibiotic resistance profiles predicted by whole-genome analysis

Whole-genome analysis identified 38 distinct ARGs among the 80 isolates. Nine ARGs were ubiquitous, including *mepA*, *mepR*, *tet* (38) (tetracyclines), *arlR*, *arlS*, *mgrA*, *norA* (fluoroquinolones), *dfrC* (sulfonamides), and *lmrS*, of which all except *dfrC* are linked to efflux-mediated resistance (55). The β-lactamase gene *blaZ* was detected in 87.5% of isolates (70/80) and frequently co-occurred with *mecA* in MRSA (*n* = 18). *fosB*, conferring fosfomycin resistance, was present in 38.8% of isolates (31/80) and significantly more common in MSSA (*P* = 0.001), primarily clustered within ST5 (*n* = 7), ST15 (*n* = 7), and ST25 (*n* = 8). *ermC* was the most predominant macrolide-lincosamide-streptogramin B (MLSB) resistance gene, found in 31 isolates (4 MRSA and 27 MSSA), mainly in ST22 (*n* = 10) and ST25 (*n* = 5) (56). The second most common variant, *ermB* (*n* = 16), mainly in ST59 (*n* = 9) and not overlapping with *ermC* presence, was significantly less frequent in MSSA than in MRSA (*P* < 0.001) (Fig. 2).

Notably, CA-MRSA-ST4513 HD54 carried a partial fragment of *bla*_OXA-485_ (577 bp, identification 99.31%; LLNM01000004, 789 bp)*,* an OXA-50-like β-lactamase gene typically associated with *Pseudomonas aeruginosa* (57, 58). HD54 also harbored *aad (6*), *aph(3')-IIIa*, *ermE*, *ermB*, *blaZ*, *mecA*, and *mexG*, in addition to the nine core ARGs. The observed resistance to cefoxitin, oxacillin, penicillin, clindamycin, and erythromycin was largely concordant with its genotypic profile. CA-MSSA-ST188 HD14 harbored *bla*_TEM-116_ (861 bp, identification 100%; NG_050162, 861 bp), a TEM-1 derivative initially identified in *Escherichia coli* and subsequently reported in various bacterial species globally (57, 59). In addition to *bla*_TEM-116_, HD14 carried *blaZ*, *ermC*, and *fosA*, partially matching phenotypic resistance to penicillin, clindamycin, and erythromycin. The XDR isolate CA-MSSA-ST398 HD146 harbored the highest number of ARGs (*n* = 19), conferring the broadest resistance profile (Fig. 2 and 4; Table S3).

#### Antibiotic treatments

Antibiotics were administered to 95.0% of patients (76/80). Most received combination regimens (68.8%, 55/80), with cephalosporins being the most prescribed (60.0%, 48/80). The five most frequently used antibiotics were cefazolin (a first-generation cephalosporin; 51.3%, 41/80), levofloxacin (42.5%, 34/80), clindamycin (25.0%, 20/80), oxacillin (18.8%, 15/80), and gentamicin (15.0%, 12/80) (Table 3).

**TABLE 3:** Antibiotic treatment for enrolled patients stratified by epidemiological subgroups^*b*^

Antibiotics	Total (*n* = 80)	Subgroups							
		CA (*n* = 48)	HA (*n* = 32)	MRSA (*n* = 19)	MSSA (*n* = 61)	CA-MRSA (*n* = 10)	HA-MRSA (*n* = 9)	CA-MSSA (*n* = 38)	HA-MSSA (*n* = 23)
Cefazolin	**41** (**51.3%**)	**26** (**54.2%**)	**15** (**46.9%**)	**9** (**47.4%**)	**32** (**52.5%**)	**5** (**50.0%**)	**4** (**44.4%**)	**21** (**55.3%**)	**11** (**47.8%**)
Cefuroxime	1 (1.3%)	^*a*^–	1 (3.1%)	–	1 (1.6%)	–	–	–	1 (4.3%)
Ceftriaxone	5 (6.3%)	3 (6.3%)	2 (6.3%)	**3** (**15.8%**)	2 (3.3%)	2 (20.0%)	1 (11.1%)	1 (2.6%)	1 (4.3%)
Cefoperazone-Sulbactam	4 (5.0%)	2 (4.2%)	2 (6.3%)	2 (10.5%)	2 (3.3%)	1 (10.0%)	1 (11.1%)	1 (2.6%)	1 (4.3%)
Oxacillin	15 (18.8%)	**9** (**18.8%**)	6 (18.8%)	**3** (**15.8%**)	12 (19.7%)	**3** (**30.0%**)	–	6 (15.8%)	**6** (**26.1%**)
Penicillin	1 (1.3%)	1 (2.0%)	–	–	1 (1.6%)	–	–	1 (2.6%)	–
Vancomycin	4 (5.0%)	2 (4.2%)	2 (6.3%)	2 (10.5%)	2 (3.3%)	1 (10.0%)	1 (11.1%)	1 (2.6%)	1 (4.3%)
Clindamycin	**20** (**25.0%**)	**9** (**18.8%**)	**11** (**31.4%**)	1 (5.3%)	**19** (**31.2%**)	1 (10.0%)	–	**8** (**21.1%**)	**11** (**47.8%**)
Erythromycin	1 (1.3%)	–	1 (3.1%)	–	1 (1.6%)	–	–	–	1 (4.3%)
Gentamicin	12 (15.0%)	6 (12.5%)	6 (18.8%)	**3** (**15.8%**)	9 (14.8%)	–	**3** (**33.3%**)	6 (15.8%)	3 (13.0%)
Ciprofloxacin	3 (3.8%)	2 (4.2%)	1 (3.1%)	1 (5.3%)	2 (3.3%)	1 (10.0%)	–	1 (2.6%)	1 (4.3%)
Levofloxacin	**34** (**42.5%**)	**25** (**52.1%**)	**9** (**28.1%**)	**12** (**63.2%**)	**22** (**36.1%**)	**8** (**80.0%**)	**4** (**44.4%**)	**17** (**44.7%**)	5 (21.7%)
Piperacillin-Tazobactam	4 (5.0%)	2 (4.2%)	2 (6.3%)	–	4 (6.6%)	–	–	2 (5.3%)	2 (8.7%)
Meropenem	1 (1.3%)	–	1 (3.1%)	1 (5.3%)	–	–	1 (11.1%)	–	–
Tinidazole	3 (3.8%)	2 (4.2%)	1 (3.1%)	–	3 (4.9%)	–	–	2 (5.3%)	1 (4.3%)

^*a*^ Not available.

^*b*^ For each subgroup, the three most frequently used antibiotics are highlighted in bold.

No statistically significant differences were observed in the distribution of patients receiving different numbers of antibiotics between CA and HA, MRSA and MSSA, or among the four subgroups (*P* = 0.249, 0.366, and 0.054, respectively). Cefazolin (54.2%, 26/48) and levofloxacin (63.2%, 12/19) were most frequently used in CA and MRSA infections, whereas cefazolin, levofloxacin, and clindamycin predominated in HA and MSSA infections. Only four patients received vancomycin, and none received linezolid (Table 3).

## DISCUSSION

This study provides an integrated clinical, phenotypic, and genetic characterization of 80 *S. aureus* isolates associated with orthopedic infections in a rural hospital in Qingdao, China. The results reveal marked diversity in genetic backgrounds and resistance determinants, highlighting the increasing threat of multidrug-resistant and virulent *S. aureus* in resource-limited settings.

*S. aureus* is one of the most significant pathogens of both hospital-acquired and community-associated infections worldwide. According to the China Antimicrobial Surveillance Network, *S. aureus* ranked as the third most frequently isolated bacterial pathogen in 2024, accounting for 9.1% of all clinical isolates. The significance of *S. aureus* in orthopedic infections, especially SSTI, has been continuously reported (60–62). Consistent with previous studies (44), *S. aureus* infections were more common among middle-aged male patients and primarily affected the lower extremities. The high proportion of surgical interventions underscores the considerable clinical and socioeconomic burden of these infections. However, due to socioeconomic constraints and limited healthcare accessibility of this cohort, many patients requested early discharge before completing full treatment courses. Consequently, the hospitalization duration analyzed here may not accurately reflect disease severity or treatment outcomes and thus has limited interpretive value across the cohort and subgroups.

While MRSA continues to draw major attention due to its significant resistance and persistence, MSSA, particularly CA-MSSA, should not be overlooked, as it has become predominant in many regions (63). Both CA- and HA-associated infections were identified in this study, with CA-MSSA being the dominant subtype. The nearly equal proportions of CA- and HA-MRSA strains further indicate the extensive circulation of resistant lineages beyond hospital boundaries.

The observed AMR patterns were alarming. Overall, 62.5% of isolates were classified as MDR, with particularly high resistance to penicillin, clindamycin, and erythromycin. All isolates remained susceptible to vancomycin and linezolid, likely reflecting their restricted clinical use. Notably, all MRSA isolates were susceptible to fluoroquinolones and gentamicin, which contrasts with typical HA-MRSA profiles. Conversely, some CA-MRSA isolates exhibited multidrug resistance resembling HA-MRSA, suggesting potential adaptation of community lineages to hospital environments.

Genomic analyses revealed a relatively diverse resistome. The prevalence of efflux-related ARGs and key determinants, such as *blaZ*, *mecA*, *ermC*, and *ermB*, along with single-copy ARGs in distinct MDR strains, aligns with previous studies and, more importantly, reflects the complex and evolving AMR landscape of *S. aureus* (64–67). The sporadic but widespread distribution of *fosB* across different collection years and both CA- and HA-origin isolates supports its mobility via plasmids and interspecies transfer (68). Its co-occurrence with *blaZ* (83.9%, 26/31), exceeding the 64.2% reported in a recent global study (69), further underscores the potential contribution of *fosB* to multidrug resistance dissemination.

The detection of *bla*_OXA-485_ and *bla*_TEM-116_ fragments, previously unreported in *S. aureus*, corroborated with ongoing interspecies β-lactamase gene transfer (59, 70). Although these fragments may not be functionally active, they warrant further investigation into their origin and mobility potential (57, 71). The discrepancies between phenotypic and genotypic AMR profiles observed here may suggest the presence of silent ARGs or alternative resistance mechanisms (72, 73). Such silent genes represent latent reservoirs that may be activated under selective pressure, facilitating unnoticed AMR transmission in clinical settings (74).

Antibiotic prescription records revealed extensive and sometimes inappropriate use. It is striking that 68.8% of the patients received two or more different drugs, while four patients had no prescription recorded. The frequent prescription of cephalosporins, clindamycin, and oxacillin might contribute to AMR acceleration, considering the fact that clindamycin and oxacillin, two of the five most commonly used antibiotics, exhibited high resistance rates (62.5% and 27.5%). Several patients received antibiotics to which their infecting pathogens were resistant. For example, the patient with clindamycin-resistant isolate HD238 was treated with clindamycin for 10 days. Three MRSA cases with confirmed oxacillin resistance were nonetheless treated with it, and one patient infected with penicillin-resistant-only HD70 received cefazolin, clindamycin, levofloxacin, and gentamicin over 78 days. Such mismatches likely accelerated resistance selection. Further, the presence of XDR strains is of particular concern, such as HD146, classified as MSSA by the clinical lab, which may have downplayed its threat, potentially delaying adequate therapy. Such extensive resistance is likely a consequence of antibiotic overuse and may severely restrict therapeutic options and prolong treatment duration (3).

Cephalosporins remain the preferred empiric agents in Chinese hospitals due to their broad spectrum and patients’ preference for rapid symptom relief (75, 76). However, penicillin and erythromycin were largely ineffective, with resistance rates of 88.8% and 63.8% in this cohort, likely reflecting their long history of overuse (77–79). Since the 2012 introduction of the “Regulations for Clinical Application of Antibacterial Agents” (80), awareness of rational antibiotic use has improved, yet stewardship challenges persist. Although agents such as vancomycin, linezolid, tigecycline, and fluoroquinolones remain effective against *S. aureus* in this study, careful monitoring is strongly recommended to prevent emerging resistance.

Systematic phenotypic and genotypic analyses revealed heterogeneity in virulence-associated traits. The predominance of β-hemolysis (77.5%) indicates enhanced cytolytic potential (81, 82). Associations between clonal pigmentation and specific STs or epidemiological subgroups were observed. Golden pigmentation in *S. aureus* is conferred by the virulence factor STX, which contributes to oxidative stress resistance. Reduced or absent pigmentation was observed in 68.8% of the isolates. However, this represents a phenotypic observation only and requires further functional validation to determine whether the STX biosynthetic pathway is inactivated or lost (45, 83). The universal catalase positivity may partially compensate for this deficiency, thereby supporting persistence in chronic or deep-seated infections (84).

Regarding other virulence, the discrepancy between phenotypic and genotypic hemolysis may reflect regulatory or environmental modulation of toxin gene expression (82). The widespread presence of IEC genes, particularly *sak* and *scn*, may contribute to infection persistence (85). The high prevalence of PVL-positive MSSA-ST22 strains (12/17) contrasts with previous Chinese studies, indicating possible regional variation or clonal replacement, warranting continuous surveillance (24, 25, 86). The observed distribution of enterotoxin-encoding genes and their association with specific STs highlights the need for larger sample sizes to draw definitive conclusions.

Notably, SCC*mec* types I–III and IV–VIII are typically associated with HA-MRSA and CA-MRSA, respectively (46, 47). However, only types IV and V were identified among both CA-MRSA and HA-MRSA isolates in this study, indicating that both SCC*mec* types can spread in community and hospital reservoirs.

Phylogenetic analyses further demonstrated that ARGs, VFs, and MGEs were enriched in an ST-dependent manner but showed no association with temporal, epidemiological, or CA/HA classification. This pattern, confirmed by integrating 515 globally reported genomes, highlights lineage-specific evolutionary trajectories of *S. aureus* in orthopedic infections.

This study has several limitations. It was conducted in a single rural hospital with a modest sample size, which may limit statistical power for detecting less prevalent sequence types, resistance determinants, or subtle phenotype and genotype associations. Healthcare parameters in rural areas, including antibiotic usage patterns, diagnostic capacity, and infection control measures, were not systematically analyzed, limiting the assessment of their impact on resistance profiles and broader application. Moreover, the study population was restricted to inpatients with orthopedic infections, which may not reflect *S. aureus* diversity circulating in the broader community and non-orthopedic infections. The cross-sectional design precludes the assessment of temporal trends, transmission dynamics, or within-host evolution of *S. aureus* over time. Resistance genes and virulence factors were identified based on genomic predictions without functional validation. In addition, the association of genetic characteristics with patient outcomes could not be analyzed due to limited long-term follow-up data that prevented robust evaluation of clinical outcomes, such as prognosis, therapy failure, or relapse. Finally, part of the study period coincided with the COVID-19 pandemic, during which strict infection control measures were implemented in both hospital and community settings in China. These policies may have influenced the local *S. aureus* transmission dynamics (87, 88). Broader, longitudinal, multicenter studies integrating genomic, clinical, and healthcare system data are warranted for a more comprehensive understanding of orthopedic *S. aureus* infections.

In summary, the current study reveals a high prevalence of MDR *S. aureus* with substantial genetic heterogeneity associated with orthopedic infections in a rural Chinese hospital. The identification of four novel STs and distinct phylogenetic clustering expands the current understanding of *S. aureus* genomic diversity and indicates ongoing local evolution. The predominance of MSSA infections further underscores its clinical relevance. These findings highlight the need for sustained surveillance, genomic monitoring, and, critically, rational antimicrobial stewardship to mitigate the burden of drug-resistant *S. aureus* in both healthcare and community settings, particularly important in resource-limited settings.

## Data Availability

All the *S. aureus* genomes generated in this study have been deposited in GenBank under BioProject PRJNA1080311 (https://www.ncbi.nlm.nih.gov/genbank/).

## SUPPLEMENTAL MATERIAL

Supplemental materials spectrum.03692-25-s0001.docx Supplemental Results, Discussion, Figures S1 and S2, and Tables S1 to S3.

ASM does not own the copyrights to Supplemental Material that may be linked to, or accessed through, an article. The authors have granted ASM a non-exclusive, world-wide license to publish the Supplemental Material files. Please contact the corresponding author directly for reuse.
