# Supplementary material for: Genomic and clinical characteristics of Staphylococcus aureus isolates associated with orthopedic infections from a rural hospital, Qingdao, China
Source: Microbiol Spectr. 2026 Mar 23;14(5):e03692-25. doi: 10.1128/spectrum.03692-25 (PMC13141964; doi:10.1128/spectrum.03692-25)
Supplement: Supplemental materials — Supplemental Results, Discussion, Figures S1 and S2, and Tables S1 to S3. [file spectrum.03692-25-s0001.docx]

Supplemental Material

Genomic and clinical characteristics of *Staphylococcus aureus* isolates associated with orthopedic infections from a rural hospital, Qingdao, China

Ying Wang,^1#†^ Yingdi Wang,^1†^ Chao Liu,^2^ Dan Zhao,^1^ Yanxiang Cui,^3^ Xiaoxuan Guan,^3^ Guilai Jiang,^4^ Shunqian Yuan,^1^ Xinting Meng,^1^ Jianqiang Hu,^5^ Wenbo Xia,^5^ Jie Liu^1#^

^1^School of Public Health, Qingdao University, Qingdao, Shandong, China

^2^Medical Department, LaiXi Municipal Hospital, Qingdao, Shandong, China

^3^Department of Clinical Laboratory, Qingdao Huangdao District Traditional Chinese Medicine Hospital, Qingdao, Shandong, China

^4^NHC Key Laboratory of Systems Biology of Pathogens, National Institute of Pathogen Biology, Chinese Academy of Medical Sciences and Peking Union Medical College, Beijing, China

^5^Department of Orthopedics, Qingdao Huangdao District Traditional Chinese Medicine Hospital, Qingdao, Shandong, China

Running Head: Genomics of *S. aureus* in orthopedic infections

^#^Address correspondence to Jie Liu, jl5yj@qdu.edu.cn; and Ying Wang, ywang0412@qdu.edu.cn.

^†^Ying Wang and Yingdi Wang contributed equally to this work. Author order was determined on the basis of seniority.

# **RESULTS AND DISCUSSION**

## **Phylogenetic analysis of the 80 *S. aureus* isolates along with publicly available genomes from the NCBI database**

The resulting phylogenetic tree revealed two major clades with uneven distribution of geographic origin and deposition time. **Clade I** comprised 13 isolates and was further divided into two subclades. Subclade I included three genomes (one from Ghana and two from Australia), all identified as ST45 and deposited in 2015. Subclade II consisted of ten genomes from four countries of three continents: seven from the U.S. (all ST45; two from 2004, five from 2014), one from Ghana (ST508, 2013), one from Tanzania (ST45, 2008), and one isolate from this study (HD169, ST3154, 2023) (Fig. 1). The ST45 dominated **Clade Ⅰ** (11/13) showed wide geographic and temporal separation, suggesting a common ancestral lineage and global dissemination of ST45. This spread may have been facilitated by international travel, medical exchanges, or other human activities (1).

**Clade Ⅱ** was subdivided into 15 subclades, within which the 79 studied isolates were unevenly distributed, with several forming tight clusters. These subclades were analyzed in detail as described below (Fig. 1).

**Clade Ⅱ-A** was divided into two branches, one of which comprised isolates from this study. This branch included 19 genomes (18 belonging to ST398) originating from six countries across four continents. These included eleven from China (collected between 2014 and 2024), three from Germany (one in 2012 and two in 2015), two from Denmark (2015), and one each from Canada (2008), Dominica (2007), and Sudan (ST140, 2017). The Chinese isolates formed a relatively tight cluster, with the Dominican genome positioned between them. This cluster extended toward the European and Canadian isolates, with HD146 (ST398, 2023) from this study and the Sudanese genome located at the outer edge.

**Clade Ⅱ-B comprised two major branches. The first branch was positioned HD112** (ST121, 2023)**, including 16 genomes originating from six countries across four continents: seven isolates from Ghana (four ST121 from 2013 and 2014, three ST3250 from 2015), three from the U.S. (one ST182 from 2009, one ST51 and one ST2276 from 2014), three from Tanzania (two ST121 and one ST2430 from 2013),** one each from Denmark (ST130, 2011), and Germany (ST182, 2013). HD112 was **phylogenetically closest** to five genomes from Ghana and Tanzania collected between 2013 and 2014, and adjacent to two genomes from the U.S. (2014) and one from Tanzania (2013).

The second branch comprised 46 genomes, including 40 from China and 6 from the U.S. Among them, 13 Chinese isolates were newly reported in this study, including ten ST59, one ST952, and two ST4513 isolates. The six American genomes included one early isolate (ST49, 1947) and five from 2014 (one ST50, two ST59, and two ST87). The remaining 27 Chinese genomes were mainly collected from Zhejiang Province in 2015 and comprised eleven ST59, eleven ST338, three ST3193, one ST5265, and one ST5267. The Chinese isolates formed a tight and coherent cluster, within which the newly reported strains were embedded. This cluster extended outward to incorporate the American isolates.

The evolutionary relatedness of ST59 strains collected in Qingdao (2021-2024) and Zhejiang (2015) suggested regional persistence and dissemination of ST59 over the past decade. Their phylogenetic proximity to American genomes, including an early 1947 isolate located at the periphery and 2014 isolates with moderate similarity, may reflect historical links or convergent evolution between Chinese and North American ST59 lineages (2).

**Clade Ⅱ-C comprised a total of 23 genomes, including 16 isolates reported in this study and collected between 2020 and 2024.** **Among these, isolate HD198 was identified as novel ST9408, and HD260 belonged to ST5461; the remaining 21 isolates were all classified as ST22. In addition, Ⅱ-C included three genomes from Kenya (2015), one each from Belgium (2011), Germany (2015), Lebanon (2016), and the U.S. (un-**specified collection year**). The Chinese isolates clustered tightly with Kenyan genomes positioned at the periphery, and the branch subsequently extended to include the European and American genomes.**

**Notably, ST22, particularly the MRSA variant, was historically rare in China but has recently shown signs of replacing dominant clones, such as ST59. Hypervirulent ST22-MRSA-IV-t309 strains have been reported to carry both PVL and TSST-1 genes, which may enhance virulence and compromise treatment outcomes (3). However, none of the ST22 isolates in this study harbored both genes, possibly due to the small sample size.**

**Clade Ⅱ-D diverged into two major branches. The first branch comprised eight genomes, including four Chinese isolates reported in this study, three European isolates (one from Germany, 2008; two from Switzerland, 2018), and one from the U.S. (2009). Among them, the Chinese isolate HD149 represented novel ST9407, while the American isolate belonged to ST1159; the remaining six genomes were classified as ST7. The Chinese and European isolates formed two distinct clusters within this branch, whereas the American isolate grouped with the Chinese cluster.**

The second branch consisted of 193 genomes collected between 1996 and 2024 from 17 countries across five continents. The earliest genome was from Japan (ST5, 1996), and the most recent were two Chinese isolates reported in this study (HD254 and HD257, both ST5, 2024). Most genomes originated from the U.S. (108) between 1999 and 2016, including seventy-two ST5 isolates and thirty ST105 isolates. Among the fifteen STs represented, ST5 was the most prevalent (74.6%, 144/193), followed by ST105 (16.1%, 31/193). ST5 isolates were recovered over a wide time span (1996-2024), with half of them from the U.S., while ST105 strains were mainly concentrated between 2003 and 2013, with 30 of 31 also from the U.S. Genomes of the same ST or geographic origin tended to cluster together, although some were intricately intertwined. Ten isolates from this study were located within this branch. Notably, HD257 (ST5, 2024) clustered closely with two Lebanese isolates (ST149, 2016). HD58 (novel ST8153, 2022), HD68 (ST59, 2022), HD106 (ST7653, 2022), and HD199 (ST5, 2023) formed a tight cluster with a Brazilian isolate (ST5, 2015). Another distinct cluster included five Chinese ST5 isolates, HD19 (2021), HD30 (2021), HD79 (2022), HD127 (2022), and HD254 (2024), together with two American ST5 genomes (2005 and 2007), one American ST5286 genome (2014), and one genome from Ghana (ST5, 2018).

The phenomenon that our ST5 isolates clustered with strains from diverse geographic regions and across a broad time span is consistent with the global dispersal and long-standing evolutionary history of ST5 (4). All our ST5 strains were MSSA, exhibited β-hemolytic activity, and carried multiple enterotoxin genes, which matched previous studies that ST5-MSSA is characterized with increased virulence (5).

**Clade Ⅱ-E** comprised 23 genomes collected between 1998 and 2024 from eight countries across five continents. Seven isolates from this study (HD14, HD23, HD70, HD133, HD178, HD180, and HD197) were all identified as ST188 and collected between 2021 and 2023. These isolates formed a tight cluster together with one from Argentina (ST188, 2005) and one from the U.S. (ST188, un-specified collection year), indicating potential global dissemination. All seven ST188 isolates were MSSA with *spa* type t189 and showed penicillin resistance, except for HD180, which remained penicillin-susceptible. This pattern is consistent with profiles previously reported in Shanghai (6). Another isolate from this study, HD248 (ST1, 2024), clustered most closely with two American ST1 genomes collected in 1998.

HD158 (ST72, 2023) was located within **Clade Ⅱ-F**, which comprised 27 genomes collected between 2008 and 2023 from seven countries spanning four continents. Most genomes were from Tanzania (n = 13) and Ghana (n = 8). ST88 was the dominant lineage within this subclade (n = 17). Phylogenetically, HD158 was most closely related to one South Korean isolate (ST72, 2014) and one Tanzanian isolate (ST3118, 2008).

**Clade Ⅱ-G** comprised 38 genomes collected between 1933 (ST464, the U.S.) and 2024 (ST15 HD211 and ST5459 HD216, the current study) from 12 countries across four continents. The two most prevalent STs in this subclade were ST15 (n = 18) and ST80 (n = 8). HD129 (ST1281, 2023) was located at the periphery of a distinct sub-branch with other eight genomes which all belonged to ST80: three from Jordan (2007, 2009), two from Lebanon (2007, 2011), and one each from Denmark (1997), Greece (2006), and the United Kingdom (2003). The other eight isolates from this study formed a tight cluster within another distinct sub-branch, which subsequently extended to connect with 12 additional genomes from Africa (Ghana, Kenya, and Tanzania), Europe (France and Germany), and the U.S. The 20 genomes of this sub-branch were collected between 2003 and 2024. Among these, 18 isolates belonged to ST15, while the German isolate was ST582 and HD216 represented ST5459. Noteworthy, our seven ST15 isolates clustered closely with strains from Africa, Europe, and North America, suggesting possible international transmission or a shared ancestral lineage with global expansion potential (7, 8).

**Clade Ⅱ-H** comprised ten genomes from this study along with two previously published French genomes (both ST25, 2009). Among the twelve genomes, ten were identified as ST25, while the remaining two, HD82 and HD126, belonged to ST8154 (new ST) and ST8621, respectively.

# **REFERENCES**

1. Effelsberg N, Stegger M, Peitzmann L, Altinok O, Coombs GW, Pichon B, Kearns A, Randad PR, Heaney CD, Bletz S, Schaumburg F, Mellmann A. 2020. Global epidemiology and evolutionary history of Staphylococcus aureus ST45. J Clin Microbiol 59(1):e02198-20.

2. McClure JA, Lakhundi S, Niazy A, Dong G, Obasuyi O, Gordon P, Chen S, Conly JM, Zhang K. 2021. Staphylococcus aureus ST59: concurrent but separate evolution of north American and east Asian lineages. Front Microbiol 12:631845.

3. Zhao HL, Wu XC, Wang BJ, Shen L, Rao LL, Wang XY, Zhang J, Xiao YH, Xu YL, Yu JY, Guo YJ, Zhou Y, Wan BS, Wu CY, Chen L, Yu FY. 2023. Phenotypic and genomic analysis of the hypervirulent ST22 methicillin-resistant Staphylococcus aureus in China. mSystems 8(3):e0124222.

4. Chen FN, Yin YY, Chen HB, Wang RB, Wang SY, Wang H. 2024. Global genetic diversity and Asian clades evolution: a phylogeographic study of Staphylococcus aureus sequence type 5. Antimicrob Agents Chemother 68(3):e0117523.

5. Jian Y, Zhao L, Zhao N, Lv HY, Liu Y, He L, Liu Q, Li M. 2021. Increasing prevalence of hypervirulent ST5 methicillin susceptible Staphylococcus aureus subtype poses a serious clinical threat. Emerg Microbes Infect 10:109-122.

6. Wang YN, Liu QY, Liu Q, Gao QQ, Lu HY, Meng HW, Xie YH, Huang Q, Ma XW, Wang H, Qin JX, Li Q, Li TM, Xia Q, Li M. 2018. Phylogenetic analysis and virulence determinant of the host-adapted Staphylococcus aureus lineage ST188 in China. Emerg Microbes Infect 7:45.

7. Donkor ES, Jamrozy D, Mills RO, Dankwah T, Amoo PK, Egyir B, Badoe EV, Twasam J, Bentley SD. 2018. A genomic infection control study for Staphylococcus aureus in two Ghanaian hospitals. Infect Drug Resist 11:1757-1765.

8. Bojang A, Chung M, Camara B, Jagne I, Guerillot R, Ndure E, Howden BP, Roca A, Ghedin E. 2024. Genomic approach to determine sources of neonatal Staphylococcus aureus infection from carriage in the Gambia. BMC Infect Dis 24:941.

**Table S1 Information on antibiotics included in this study.**

| **Antibiotics** | **Drug Classes** | **Targets** | **Mechanism of action (MOA)** |
| --- | --- | --- | --- |
| Cefoxitin | Cephalosporins (2^nd^ generation) | Cell envelope | Inhibition of cell envelope synthesis |
| Oxacillin | Penicillins | Cell envelope | Inhibition of cell envelope synthesis |
| Penicillin | Penicillins | Cell envelope | Inhibition of cell envelope synthesis |
| Vancomycin | Glycopeptides | Others | Inhibition of cell envelope synthesis |
| Clindamycin | Lincosamides | 50S ribosome subunit | Inhibition of protein synthesis |
| Erythromycin | Macrolides | 50S ribosome subunit | Inhibition of protein synthesis |
| Gentamicin | Aminoglycosides | 30S ribosome subunit | Inhibition of protein synthesis |
| Linezolid | Oxazolidinones | 50S ribosome subunit | Inhibition of protein synthesis |
| Quinupristin/Dalfopristin | Streptogramins | 50S ribosome subunit | Inhibition of protein synthesis |
| Tetracycline | Tetracyclines | 30S ribosome subunit | Inhibition of protein synthesis |
| Tigecycline | Glycylcycline | 30S ribosome subunit | Inhibition of protein synthesis |
| Ciprofloxacin | Fluoroquinolones | DNA topoisomerases | Inhibition of DNA synthesis |
| Levofloxacin | Fluoroquinolones | DNA topoisomerases | Inhibition of DNA synthesis |
| Moxifloxacin | Fluoroquinolones | DNA topoisomerases | Inhibition of DNA synthesis |
| Rifampicin | Ansamycins | Others | Drugs against mycobacteria, inhibiting transcription |
| Sulfamethoxazole | Sulfonamides | Others | Others (blocking the microorganisms to make and to use folate) |

**Table S2 Genotypic and phenotypic characterization of the 80 *S. aureus* isolates.**

| **Isolates** | **Collection date** | **CC*^a^*** | **ST*^b^*** | ***spa*** | **SCC*mec*** | **Subgroups** | **Clonal**  **pigmentation** | **Hemolytic types** | **Catalase activity** |
| --- | --- | --- | --- | --- | --- | --- | --- | --- | --- |
| HD1 | 201227*^c^* | CC22 | ST22 | t309 | V | HA-MRSA | White | β | ＋*^d^* |
| HD9 | 210203 | CC25 | ST25 | t78 | NA | HA-MSSA | White | β | ＋ |
| HD14 | 210808 | CC1 | ST188 | t189 | NA | CA-MSSA | Yellow | α | ＋ |
| HD19 | 210603 | CC5 | ST5 | t2 | NA | HA-MSSA | White | β | ＋ |
| HD23 | 210327 | CC1 | ST188 | t189 | NA | HA-MSSA | White | β | ＋ |
| HD28 | 210726 | CC398 | ST398 | t1451 | NA | HA-MSSA | White | β | ＋ |
| HD30 | 210817 | CC5 | ST5 | t67 | NA | HA-MSSA | Yellow | β | ＋ |
| HD34 | 211101 | CC25 | ST25 | t78 | NA | HA-MSSA | White | α | ＋ |
| HD38 | 211112 | CC398 | ST398 | t1451 | NA | CA-MSSA | White | β | ＋ |
| HD43 | 220110 | CC59 | ST59 | t437 | IVa | HA-MRSA | White | β | ＋ |
| HD50 | 210926 | CC25 | ST25 | t78 | NA | HA-MSSA | White | β | ＋ |
| HD54 | 211009 | unknown | ST4513 | t437 | IVa | CA-MRSA | Yellow | α | ＋ |
| HD57 | 220219 | CC25 | ST25 | t78 | NA | CA-MSSA | White | β | ＋ |
| HD58 | 220407 | CC5 | ST8153 | t586 | NA | HA-MSSA | Yellow | β | ＋ |
| HD59 | 220331 | CC59 | ST59 | t441 | IVa | CA-MRSA | Yellow | β | ＋ |
| HD60 | 220512 | CC59 | ST59 | t437 | IVa | HA-MRSA | Yellow | α | ＋ |
| HD67 | 220627 | CC59 | ST59 | t3736 | IVa | CA-MRSA | Yellow | α | ＋ |
| HD68 | 220407 | CC59 | ST59 | t954 | NA | CA-MSSA | Yellow | β | ＋ |
| HD69 | 220603 | CC15 | ST15 | t2325 | NA | CA-MSSA | White | β | ＋ |
| HD70 | 220302 | CC1 | ST188 | t189 | NA | CA-MSSA | Yellow | β | ＋ |
| HD73 | 220506 | unknown | ST7 | t796 | NA | CA-MSSA | White | β | ＋ |
| HD74 | 220505 | CC398 | ST398 | unknown | NA | HA-MSSA | White | α | ＋ |
| HD77 | 220729 | CC22 | ST22 | t309 | NA | HA-MSSA | White | β | ＋ |
| HD79 | 220404 | CC5 | ST5 | t2 | NA | CA-MSSA | White | β | ＋ |
| HD81 | 220503 | CC15 | ST15 | t346 | NA | CA-MSSA | White | β | ＋ |
| HD82 | 220323 | unknown | ST8154 | t5355 | NA | HA-MSSA | White | γ | ＋ |
| HD106 | 220327 | CC5 | ST7653 | t954 | NA | CA-MSSA | Yellow | α | ＋ |
| HD112 | 230217 | CC121 | ST121 | t2019 | NA | CA-MSSA | Yellow | α | ＋ |
| HD116 | 220903 | CC22 | ST22 | t309 | NA | CA-MSSA | Yellow | β | ＋ |
| HD121 | 220911 | CC25 | ST25 | t78 | NA | HA-MSSA | White | β | ＋ |
| HD126 | 220930 | unknown | ST8621 | unknown | NA | HA-MSSA | White | β | ＋ |
| HD127 | 221022 | CC5 | ST5 | t9228 | NA | CA-MSSA | Yellow | β | ＋ |
| HD129 | 230227 | unknown | ST1281 | t164 | NA | CA-MSSA | White | γ | ＋ |
| HD130 | 221130 | CC398 | ST398 | unknown | V | CA-MRSA | White | β | ＋ |
| HD133 | 221128 | CC1 | ST188 | t189 | NA | CA-MSSA | White | α | ＋ |
| HD137 | 221009 | CC15 | ST15 | t84 | NA | CA-MSSA | Yellow | α | ＋ |
| HD141 | 230211 | CC22 | ST22 | t15791 | NA | CA-MSSA | White | β | ＋ |
| HD143 | 220818 | CC22 | ST22 | t1977 | NA | CA-MSSA | White | β | ＋ |
| HD145 | 220818 | CC22 | ST22 | t309 | NA | CA-MSSA | Yellow | β | ＋ |
| HD146 | 230304 | CC398 | ST398 | t1451 | NA | CA-MSSA | White | γ | ＋ |
| HD149 | 221002 | unknown | ST9407 | t18926 | NA | CA-MSSA | White | β | ＋ |
| HD150 | 230417 | CC59 | ST59 | t1212 | IVa | CA-MRSA | Yellow | β | ＋ |
| HD151 | 230425 | CC22 | ST22 | t309 | NA | CA-MSSA | White | β | ＋ |
| HD153 | 230326 | CC59 | ST59 | t437 | IVa | HA-MRSA | White | β | ＋ |
| HD154 | 230714 | CC59 | ST59 | t437 | IVa | HA-MRSA | Yellow | β | ＋ |
| HD158 | 230718 | CC8 | ST72 | unknown | IVc | HA-MRSA | White | β | ＋ |
| HD160 | 230723 | CC15 | ST15 | t84 | NA | HA-MSSA | Yellow | β | ＋ |
| HD162 | 230419 | CC15 | ST15 | t3698 | NA | HA-MSSA | White | β | ＋ |
| HD163 | 230324 | CC15 | ST15 | t12513 | NA | CA-MSSA | Yellow | β | ＋ |
| HD169 | 230908 | CC45 | ST3154 | unknown | NA | CA-MSSA | White | α | ＋ |
| HD171 | 231001 | CC59 | ST59 | t8347 | IVa | HA-MRSA | White | α | ＋ |
| HD177 | 231008 | CC59 | ST59 | t437 | NA | CA-MSSA | White | β | ＋ |
| HD178 | 231014 | CC1 | ST188 | t189 | NA | HA-MSSA | White | β | ＋ |
| HD180 | 230825 | CC1 | ST188 | t189 | NA | HA-MSSA | White | β | ＋ |
| HD183 | 231002 | CC25 | ST25 | t1521 | NA | CA-MSSA | White | α | ＋ |
| HD185 | 230913 | CC25 | ST25 | t78 | NA | HA-MSSA | White | β | ＋ |
| HD191 | 230801 | unknown | ST7 | t91 | IV | HA-MRSA | White | α | ＋ |
| HD196 | 230920 | CC398 | ST398 | unknown | V | HA-MRSA | White | β | ＋ |
| HD197 | 231205 | CC1 | ST188 | t189 | NA | CA-MSSA | White | β | ＋ |
| HD198 | 231216 | unknown | ST9408 | t309 | V | CA-MRSA | White | β | ＋ |
| HD199 | 231205 | CC5 | ST5 | t688 | NA | CA-MSSA | White | β | ＋ |
| HD201 | 231202 | CC22 | ST22 | unknown | V | CA-MRSA | White | β | ＋ |
| HD202 | 231203 | CC25 | ST25 | t287 | NA | HA-MSSA | White | β | ＋ |
| HD211 | 240428 | CC15 | ST15 | t346 | NA | HA-MSSA | White | β | ＋ |
| HD216 | 240428 | CC15 | ST5459 | t11579 | NA | CA-MSSA | White | β | ＋ |
| HD222 | 240313 | CC22 | ST22 | t13828 | NA | CA-MSSA | White | β | ＋ |
| HD223 | 240122 | CC22 | ST22 | unknown | NA | CA-MSSA | White | β | ＋ |
| HD230 | 240113 | CC22 | ST22 | unknown | NA | CA-MSSA | White | β | ＋ |
| HD238 | 240621 | unknown | ST7 | t91 | NA | HA-MSSA | White | β | ＋ |
| HD240 | 240705 | CC22 | ST22 | t309 | NA | CA-MSSA | White | β | ＋ |
| HD241 | 240621 | CC22 | ST22 | t309 | V | CA-MRSA | White | β | ＋ |
| HD248 | 240717 | CC1 | ST1 | t127 | NA | HA-MSSA | White | β | ＋ |
| HD249 | 240731 | CC59 | ST59 | t441 | NA | CA-MSSA | Yellow | β | ＋ |
| HD253 | 240819 | CC398 | ST398 | unknown | NA | CA-MSSA | White | α | ＋ |
| HD254 | 240728 | CC5 | ST5 | t11657 | NA | CA-MSSA | Yellow | β | ＋ |
| HD257 | 240820 | CC5 | ST5 | t7348 | NA | CA-MSSA | Yellow | β | ＋ |
| HD259 | 240716 | unknown | ST4513 | t437 | IVa | CA-MRSA | Yellow | β | ＋ |
| HD260 | 240804 | CC22 | ST5461 | t541 | NA | CA-MSSA | Yellow | β | ＋ |
| HD280 | 240906 | CC22 | ST22 | t309 | NA | HA-MSSA | White | β | ＋ |
| HD287 | 241028 | unknown | ST952 | t3736 | Vb | CA-MRSA | Yellow | β | ＋ |

*^a^*CC: clonal complex.

*^b^*ST: sequence type.

*^c^*Collection date 201227 indicates December 27, 2020, with subsequent dates interpreted in the same way.

*^d^*The symbol ‘+’ denotes a catalase-positive result.

**Table S3 Antimicrobial resistance profiles of the 80 *S. aureus* isolates.**

|  | **Inhibition of cell envelope synthesis** | | | **Inhibition of protein synthesis** | | | | **Inhibition of DNA synthesis** | | | **Others** |
| --- | --- | --- | --- | --- | --- | --- | --- | --- | --- | --- | --- |
| **Isolates** | **Cephalosporins** | **Penicillins** | | **Lincosamides** | **Macrolides** | **Aminoglycosides** | **Tetracyclines** | **Fluoroquinolones** | | | **Sulfonamides** |
| HD1 | Cefoxitin | Oxacillin | Penicillin | - | - | - | - | - | - | - | - |
| HD9 | -*^b^* | - | Penicillin | - | - | - | Tetracycline | - | - | - | - |
| HD14*^a^* | - | - | Penicillin | Clindamycin | Erythromycin | - | - | - | - | - | - |
| HD19*^a^* | - | - | Penicillin | Clindamycin | Erythromycin | Gentamicin | - | - | - | - | Sulfamethoxazole |
| HD23 | - | - | Penicillin | - | - | - | - | - | - | - | - |
| HD28*^a^* | - | - | Penicillin | Clindamycin | Erythromycin | - | - | - | - | - | - |
| HD30*^a^* | - | - | Penicillin | Clindamycin | Erythromycin | Gentamicin | Tetracycline | - | - | - | Sulfamethoxazole |
| HD34*^a^* | - | Oxacillin | Penicillin | Clindamycin | Erythromycin | - | - | - | - | - | - |
| HD38 | - | - | - | - | - | - | - | - | - | - | - |
| HD43*^a^* | Cefoxitin | Oxacillin | Penicillin | Clindamycin | Erythromycin | - | Tetracycline | - | - | - | - |
| HD50*^a^* | - | - | Penicillin | Clindamycin | Erythromycin | - | - | - | - | - | Sulfamethoxazole |
| HD54*^a^* | Cefoxitin | Oxacillin | Penicillin | Clindamycin | Erythromycin | - | - | - | - | - | - |
| HD57*^a^* | - | - | Penicillin | Clindamycin | Erythromycin | - | - | Ciprofloxacin | Levofloxacin | - | Sulfamethoxazole |
| HD58 | - | - | - | - | - | - | - | - | - | - | - |
| HD59*^a^* | Cefoxitin | Oxacillin | Penicillin | Clindamycin | Erythromycin | - | Tetracycline | - | - | - | - |
| HD60*^a^* | Cefoxitin | Oxacillin | Penicillin | - | - | - | Tetracycline | - | - | - | - |
| HD67*^a^* | Cefoxitin | Oxacillin | Penicillin | Clindamycin | Erythromycin | - | Tetracycline | - | - | - | - |
| HD68*^a^* | - | - | Penicillin | Clindamycin | Erythromycin | - | - | Ciprofloxacin | Levofloxacin | Moxifloxacin | - |
| HD69*^a^* | - | - | Penicillin | Clindamycin | Erythromycin | - | - | - | - | - | - |
| HD70 | - | - | Penicillin | - | - | - | - | - | - | - | - |
| HD73*^a^* | - | - | Penicillin | Clindamycin | Erythromycin | Gentamicin | - | - | - | - | Sulfamethoxazole |
| HD74 | - | - | - | Clindamycin | Erythromycin | - | - | - | - | - | - |
| HD77 | - | - | Penicillin | - | - | - | - | - | - | - | - |
| HD79*^a^* | - | - | Penicillin | Clindamycin | Erythromycin | Gentamicin | - | - | - | - | Sulfamethoxazole |
| HD81 | - | - | Penicillin | - | - | - | - | - | - | - | - |
| HD82*^a^* | - | - | Penicillin | Clindamycin | Erythromycin | - | Tetracycline | - | - | - | - |
| HD106*^a^* | - | - | Penicillin | Clindamycin | Erythromycin | - | - | Ciprofloxacin | Levofloxacin | Moxifloxacin | - |
| HD112 | - | - | Penicillin | - | - | - | - | - | - | - | Sulfamethoxazole |
| HD116*^a^* | - | Oxacillin | Penicillin | Clindamycin | Erythromycin | - | - | - | - | - | - |
| HD121 | - | - | Penicillin | - | - | - | Tetracycline | - | - | - | Sulfamethoxazole |
| HD126*^a^* | - | - | Penicillin | Clindamycin | Erythromycin | - | - | - | - | - | - |
| HD127*^a^* | - | - | - | Clindamycin | Erythromycin | - | - | Ciprofloxacin | Levofloxacin | - | Sulfamethoxazole |
| HD129 | - | - | Penicillin | - | - | - | - | - | - | - | - |
| HD130*^a^* | Cefoxitin | Oxacillin | Penicillin | Clindamycin | Erythromycin | - | - | - | - | - | - |
| HD133 | - | - | Penicillin | - | - | - | - | - | - | - | - |
| HD137*^a^* | - | - | Penicillin | Clindamycin | Erythromycin | - | - | - | - | - | - |
| HD141*^a^* | - | - | Penicillin | Clindamycin | Erythromycin | - | - | - | - | - | - |
| HD143*^a^* | - | - | Penicillin | Clindamycin | Erythromycin | - | - | - | - | - | - |
| HD145*^a^* | - | - | Penicillin | Clindamycin | Erythromycin | - | - | - | - | - | - |
| HD146*^a^* | - | - | Penicillin | Clindamycin | Erythromycin | - | Tetracycline | Ciprofloxacin | Levofloxacin | Moxifloxacin | Sulfamethoxazole |
| HD149 | - | - | Penicillin | - | - | - | - | - | - | - | - |
| HD150*^a^* | Cefoxitin | Oxacillin | Penicillin | Clindamycin | Erythromycin | - | - | - | - | - | - |
| HD151*^a^* | - | - | Penicillin | Clindamycin | Erythromycin | - | - | - | - | - | - |
| HD153*^a^* | Cefoxitin | Oxacillin | Penicillin | Clindamycin | Erythromycin | - | - | - | - | - | - |
| HD154*^a^* | Cefoxitin | - | Penicillin | Clindamycin | Erythromycin | - | Tetracycline | - | - | - | - |
| HD158*^a^* | Cefoxitin | Oxacillin | Penicillin | - | - | - | Tetracycline | - | - | - | - |
| HD160*^a^* | - | - | Penicillin | Clindamycin | Erythromycin | Gentamicin | - | Ciprofloxacin | Levofloxacin | Moxifloxacin | - |
| HD162 | - | - | Penicillin | - | - | - | - | - | - | - | - |
| HD163 | - | - | - | - | - | - | - | - | - | - | - |
| HD169*^a^* | - | - | Penicillin | Clindamycin | Erythromycin | - | - | - | - | - | - |
| HD171*^a^* | Cefoxitin | Oxacillin | Penicillin | Clindamycin | Erythromycin | - | - | - | - | - | - |
| HD177*^a^* | - | - | Penicillin | Clindamycin | Erythromycin | - | - | - | - | - | - |
| HD178 | - | - | Penicillin | - | - | - | - | - | - | - | - |
| HD180 | - | - | - | - | - | - | - | - | - | - | - |
| HD183*^a^* | - | Oxacillin | Penicillin | Clindamycin | Erythromycin | - | - | - | - | - | Sulfamethoxazole |
| HD185*^a^* | - | - | Penicillin | Clindamycin | Erythromycin | - | - | - | - | - | Sulfamethoxazole |
| HD191 | Cefoxitin | Oxacillin | Penicillin | - | - | - | - | - | - | - | - |
| HD196 | Cefoxitin | Oxacillin | Penicillin | - | - | - | - | - | - | - | - |
| HD197 | - | - | Penicillin | - | - | - | - | - | - | - | - |
| HD198*^a^* | Cefoxitin | Oxacillin | Penicillin | Clindamycin | Erythromycin | - | - | - | - | - | - |
| HD199 | - | - | Penicillin | - | - | - | - | - | - | - | - |
| HD201*^a^* | Cefoxitin | Oxacillin | Penicillin | Clindamycin | Erythromycin | - | - | - | - | - | - |
| HD202 | - | - | Penicillin | - | - | - | - | - | - | - | - |
| HD211 | - | - | Penicillin | - | Erythromycin | - | - | - | - | - | - |
| HD216*^a^* | - | - | Penicillin | Clindamycin | Erythromycin | - | - | - | - | - | - |
| HD222 | - | - | Penicillin | - | - | - | - | - | - | - | - |
| HD223*^a^* | - | - | Penicillin | Clindamycin | Erythromycin | - | - | - | - | - | - |
| HD230 | - | - | - | Clindamycin | Erythromycin | - | - | - | - | - | - |
| HD238*^a^* | - | - | Penicillin | Clindamycin | - | - | Tetracycline | - | - | - | - |
| HD240 | - | - | Penicillin | - | - | - | - | - | - | - | - |
| HD241*^a^* | Cefoxitin | Oxacillin | Penicillin | Clindamycin | Erythromycin | - | - | - | - | - | - |
| HD248*^a^* | - | - | Penicillin | - | Erythromycin | - | - | - | - | - | Sulfamethoxazole |
| HD249*^a^* | - | Oxacillin | Penicillin | Clindamycin | Erythromycin | - | - | - | - | - | - |
| HD253 | - | - | Penicillin | - | - | - | - | - | - | - | - |
| HD254 | - | - | - | - | - | - | - | Ciprofloxacin | Levofloxacin | Moxifloxacin | - |
| HD257 | - | - | - | Clindamycin | Erythromycin | - | - | - | - | - | - |
| HD259*^a^* | Cefoxitin | Oxacillin | Penicillin | Clindamycin | Erythromycin | - | Tetracycline | - | - | - | - |
| HD260*^a^* | - | - | Penicillin | Clindamycin | Erythromycin | - | - | - | - | - | - |
| HD280*^a^* | - | - | Penicillin | Clindamycin | Erythromycin | - | - | - | - | - | - |
| HD287*^a^* | Cefoxitin | Oxacillin | Penicillin | Clindamycin | Erythromycin | - | - | - | - | - | - |
| MDR*^c^* | 16 | 19 | 49 | 47 | 47 | 5 | 11 | 6 | 6 | 4 | 11 |
| Resistance*^d^* | 19 | 22 | 71 | 50 | 51 | 5 | 13 | 7 | 7 | 5 | 13 |
| Proportion*^e^* | 84.2% | 86.4% | 69.0% | 94.0% | 92.2% | 100.0% | 84.6% | 85.7% | 85.7% | 80.0% | 84.6% |

*^a^*Multidrug-resistant (MDR) strains.

*^b^*Not available.

*^c^*Number of MDR isolates resistant to each tested antibiotic.

*^d^*Number of isolates resistant to each tested antibiotic.

*^e^*The percentage of MDR isolates among all resistant strains.

**
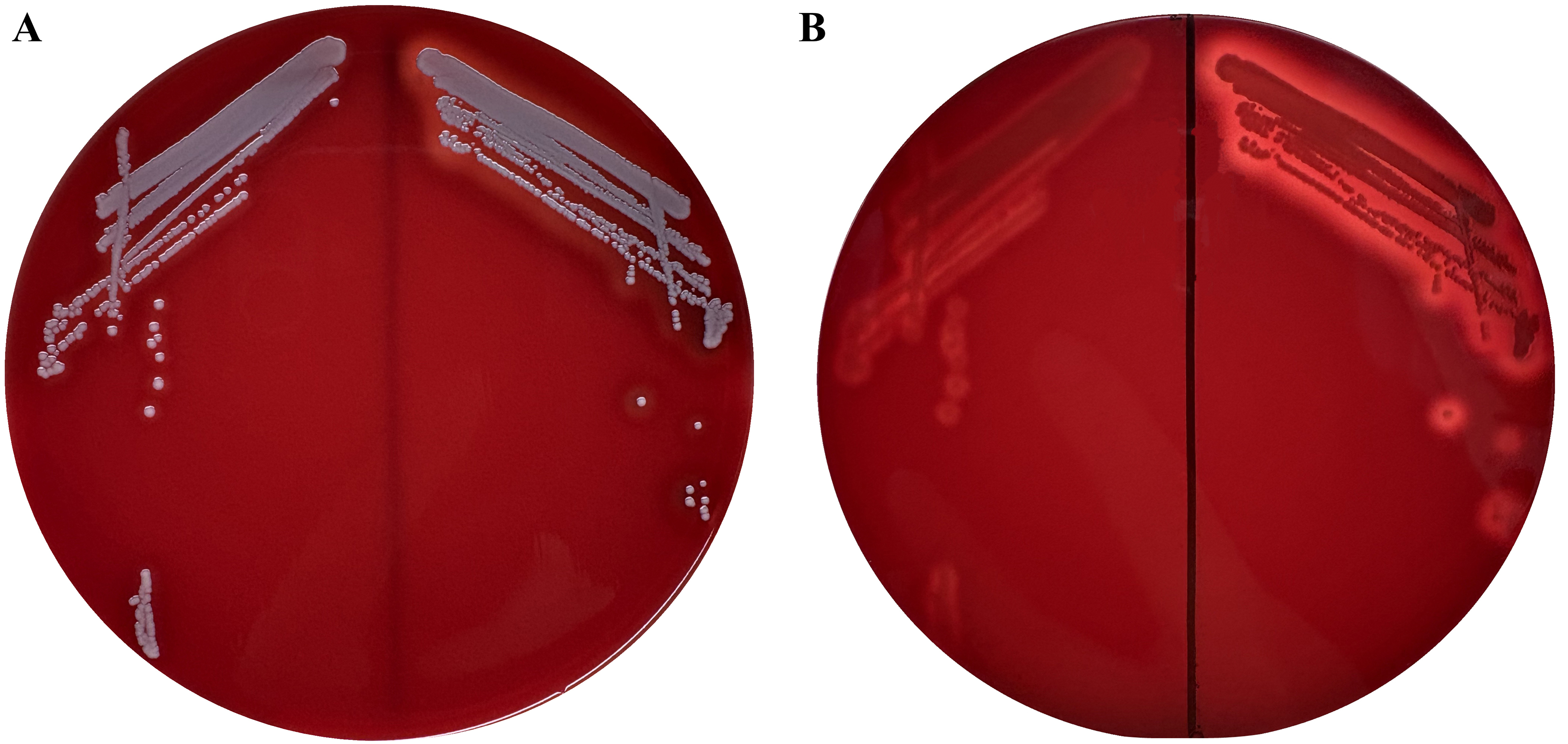
**

**Figure S1 Representative images showing α-hemolysis (left side of the plate) and β-hemolysis (right side of the plate) on blood agar. A** shows the front (surface) view of the plate, and **B** shows the corresponding view from the bottom.

**

**

**Figure S2 Distribution of the 80 *S. aureus* isolates by sequence types (STs) and epidemiological subgroups. A** Number of isolates corresponding to each detected ST. **B** Temporal distribution of CA-MRSA, HA-MRSA, CA-MSSA, and HA-MSSA isolates during the study period.
